# Cocaine craving and use during pharmacotherapy trials for cocaine use disorder: A multi-trajectory analysis

**DOI:** 10.1016/j.drugalcdep.2025.112841

**Published:** 2025-08-15

**Authors:** Ramin Mojtabai, Ryoko Susukida, Mehdi Farokhnia, Trang Quynh Nguyen, Lorenzo Leggio, Cecilia Bergeria, Kelly E. Dunn, Masoumeh Aminesmaeili

**Affiliations:** a Department of Psychiatry and Behavioral Sciences, Tulane Medical School, New Orleans, LA, USA; b Department of Mental Health, Johns Hopkins Bloomberg School of Public Health, Baltimore, MD, USA; c Clinical Psychoneuroendocrinology and Neuropsychopharmacology Section, Translational Addiction Medicine Branch, National Institute on Drug Abuse Intramural Research Program and National Institute on Alcohol Abuse and Alcoholism Division of Intramural Clinical and Biological Research, National Institutes of Health, Bethesda, MD, USA; d Department of Psychiatry and Behavioral Sciences, Johns Hopkins School of Medicine, Baltimore, MD, USA; e Kahlert Institute for Addiction Medicine, University of Maryland School of Medicine, Baltimore, MD, USA; f Iranian National Center for Addiction Studies (INCAS), Tehran University of Medical Sciences, Tehran 1336616357, Iran

**Keywords:** Cocaine use disorder, Stimulants, Craving, Recovery, Secondary data analysis, Non-abstinence outcomes, Harm reduction

## Abstract

**Background:**

There is limited research on the course of drug craving in treatment trials of stimulant use disorders. This study examined trajectories of cocaine craving and use and their associations with other outcomes of cocaine use disorder in pharmacotherapy trials of cocaine use disorder.

**Methods:**

In 1070 participants from 6 randomized controlled trials testing selegiline, baclofen, cabergoline, modafinil, reserpine, and tiagabine, we used multi-trajectory modeling to identify joint trajectories of weekly-measured cocaine craving and use. Association of these trajectories with Addiction Severity Index (ASI) health and social outcomes was assessed.

**Results:**

A 3-trajectory model with High craving/High use (40.0 %), Decreasing craving/High use (36.8 %), and Decreasing craving/Decreasing use (23.2 %) groups was the most parsimonious. Compared to the High craving/High use group, the Decreasing craving/Decreasing use group experienced significantly greater improvement on ASI domains of drug use (change score = −13.7 vs. −3.3), alcohol use (−7.3 vs. −4.4), psychiatric status (−5.6 vs. 0.7) and relationships status (−7.0 vs. −2.8) (all p < 0.05). Compared to placebo, those on modafinil 200 mg/day were more likely to be in the Decreasing craving/Decreasing use group (Relative Risk Ratio [RRR]=4.84, 95 % CI=1.38–17.00) or Decreasing craving/High use group (RRR=5.55, 2.04–14.08) than in the High craving/High use group.

**Conclusions:**

Trajectories of craving/drug use in clinical trials for cocaine use disorder are heterogeneous. Participants experiencing the greatest reduction in cocaine craving/use experienced the greatest improvement in other measures of drug use and psychosocial functioning, supporting the utility of reduced craving/use as a clinically relevant outcome in pharmacotherapy trials of people with cocaine use disorder.

## 1. Introduction

Craving is a core component and an important clinical feature in people with substance use disorders. Indeed, craving was added as one of the diagnostic criteria of substance use disorders, including stimulant use disorders, in the fifth edition of the *Diagnostic and Statistical Manual of Mental Disorders* (DSM-5), partly based on the view that craving “may become a biological treatment target” (Hasin et al., 2013). Past research suggests that craving is intrinsically associated with other outcomes of stimulant use disorder, including return to use and remission (Ahmadi et al., 2019; Gawin and Kleber, 1984; Huang et al., 2023; Kampman et al., 2015; Kosten et al., 1987; Margolin et al., 1991; Newton et al., 2006; Preston et al., 2009; Ray et al., 2015; Trivedi et al., 2021).

Pharmacotherapy trials for stimulant use disorders often select candidate medications based on their potential anti-craving properties (Lassi et al., 2022). Yet, with few exceptions (Galloway et al., 2009; Hartz et al., 2001; Hillhouse et al., 2007), studies have not specifically examined the association of the craving trajectories with trajectories of drug use or other relevant outcomes in the course of treatment trials. Furthermore, while individual responses to experimental pharmacotherapies for stimulant use disorders are known to be highly variable (Ma et al., 2013; Soares and Pereira, 2019; Zorick et al., 2011), possible heterogeneity in the course of craving during pharmacotherapy trials for stimulant use disorder has not been examined. Such heterogeneity may partly explain the negative or inconclusive results of pharmacotherapy trials (for reviews see for example: Chan et al., 2019; Lassi et al., 2022).

In a previous study using pooled data from 5 randomized controlled trials (RCTs) of pharmacotherapies for methamphetamine use disorder, we identified 3 trajectories of craving (Mojtabai et al., 2024). These trajectories were strongly correlated with trajectories of methamphetamine use in the course of the RCTs and with other outcomes at the end of the RCTs. The group experiencing the greatest reduction in craving also experienced the greatest reduction in drug use and the greatest improvement in other health-related outcomes. However, the study was specifically focused on methamphetamine use disorder RCTs, and the trajectories of craving and drug use were not jointly modeled.

In the present study, we conducted secondary analyses of pooled data from 6 pharmacotherapy RCTs for cocaine use disorder to examine trajectories of cocaine craving and use and their associations with other outcomes such as psychosocial functioning. We further examined predictors of the joint craving and cocaine use trajectories, including medications used in the RCTs and baseline characteristics of participants. We expanded upon our previous study (Mojtabai et al., 2024) by conducting multi-trajectory modeling of craving and cocaine use and by examining trajectories in cocaine use assessed by urine toxicology as well as self-report.

## 2. Methods

### 2.1. Sample

Data were drawn from a harmonized dataset of 6 National Institute on Drug Abuse (NIDA)-sponsored RCTs of pharmacotherapies for cocaine use disorder. Eligibility criteria for all RCTs included current cocaine dependence according to the DSM-IV (American Psychiatric Association, 1994) criteria. Pharmacotherapies included the monoamine oxidase inhibitor selegiline (Elkashef et al., 2006), the gamma-aminobutyric acid-B (GABA-B) receptor agonist baclofen (Kahn et al., 2009), the dopamine D2 agonist cabergoline (Shoptow et al., 2025), the non-amphetamine stimulant modafinil (Anderson et al., 2009), the vesicular monoamine transporter blocker reserpine (Winhusen et al., 2007b), and the GABA reuptake inhibitor tiagabine (Winhusen et al., 2007a). All RCTs had a placebo arm, were double-blind, and all participants received cognitive behavioral therapy as an adjunct. A brief description of these studies is provided in Supplement A.

These studies were selected from a pool of 18 pharmacotherapy RCTs for cocaine use disorder available on the NIDA Data Share site (https://datashare.nida.nih.gov/), accessed on April 5, 2023. Studies were selected if they were (1) efficacy RCTs comparing an active medication with a placebo for treatment of cocaine use disorder and (2) included the Brief Substance Craving Scale (BSCS). Pilot studies and studies examining safety and drug interactions were not included (Supplement B). The analyzed RCTs employed similar designs and collected almost identical measurements, justifying pooling of their samples.

Methods used for harmonization of these RCT data have been previously described (Susukida et al., 2020a). Briefly, all core measures, including demographic data, psychosocial measures, craving assessments, Addiction Severity Index (ASI) ratings, and measures of drug use were harmonized across RCTs for pooled analyses. The primary RCTs were approved by the respective institutions’ ethical boards, and this analysis was deemed exempt from review by the Tulane University Institutional Review Board.

### 2.2. Measures

*Cocaine craving* was assessed 3 times a week using the multi-item BSCS, administered in all 6 RCTs. BSCS was originally derived from the drug craving section of the *State of Feelings and Cravings Questionnaire* (Mezinskis et al., 1998, 2001). The questions in BSCS ask about the intensity, frequency, and duration of craving for the primary and secondary drugs in the past 24 h. For this study, we only used BSCS ratings for the primary drug (cocaine). Intensity was measured on a Likert scale ranging from “not at all” (0) to “extreme” (4); frequency was measured on a scale from “never” (0) to “almost constantly” (4); and duration was measured on a scale from “none at all” (0) to “very long” (4). As per the standard practice, the scores on the 3 items were summed up to compute a BSCS summary score ranging from 0 to 12 (Leiderman et al., 2005). The internal consistency and construct validity of the BSCS and its sensitivity to treatment effect were established in past research (Adler et al., 2010; Elkashef et al., 2005; Mezinskis et al., 1998, 2001). The mean craving rating for each week was computed by averaging across the ratings in that week.

*Urine-toxicology (Utox) ascertained cocaine use* was assessed 3 times a week in all 6 RCTs. For this study, Utox-ascertained cocaine use was operationalized as *any* positive test result for the drug (qualitative) during the week.

*Self-reported cocaine use* was assessed using the *Timeline Followback (TLFB)* (Robinson et al., 2014), also administered 3 times a week in all 6 RCTs to retrospectively assess drug use since the last assessment. Similar to Utox-ascertained use, self-reported cocaine use was operationalized as *any* use during the week.

Severity of any drug or alcohol use, as well as psychiatric, medical, legal, employment, and relationships status at baseline and at the last available assessment point of the RCT, were evaluated using the ASI. Functioning in each of these domains was measured on a scale ranging from 0 to 1, with higher scores representing worse functioning. Questions on ASI cover the past 30 days. ASI is a widely used clinical research measure with well-established reliability and validity (McLellan et al., 1985). We have previously established the validity of the ASI psychiatric domain against diagnoses of mental disorders based on semi-structured interviews (Susukida et al., 2020b). ASI drug use scale captures the extent of use of drugs besides the primary drug, history of treatment, distress associated with drug use, and self-rating of the importance of treatment for drug use.

Use of any other drugs (including heroin, methadone, other opioids/analgesics, barbiturates, sedative/hypnotic/tranquilizers, amphetamines, hallucinogens, and inhalants), injection drug use in the past 30 days (yes/no), and lifetime duration of cocaine use in years were also assessed and recorded.

Sociodemographic characteristics, including sex, age, and race/ethnicity, were assessed using a standardized demographic questionnaire.

### 2.3. Statistical analysis

An extension of the group-based trajectory modeling (GBTM) specifically designed for analyses of multiple trajectories, named *group-based multi-trajectory modeling,* was used to identify groups of participants based on trajectories of craving, Utox-ascertained cocaine use, and self-reported cocaine use in the course of RCTs (Girard et al., 2019; Nagin et al., 2018). Multi-trajectory modeling allows for parsimonious analysis of groups that are based on the joint trajectories of more than 1 outcome. Trajectory group membership is assigned probabilistically (based on posterior probabilities).

Analyses were conducted in separate stages as recommended for multi-trajectory modeling (Girard et al., 2019; Nagin et al., 2018). First, the number of trajectory groups was identified based on fit indices, indicators of classification adequacy, as well as parsimony and comprehensibility of the model (Nagin, 2005). Fit indices included the Bayesian Information Criterion (BIC) and Akaike Information Criterion (AIC). The Guidelines for Reporting on Latent Trajectory Studies recommends using BIC for selecting the number of groups (Van De Schoot et al., 2017). Larger (less negative) BIC and AIC values in GBTM models indicate a better fit of the data to the model (Nagin, 2005). Two BIC estimates were computed, one for the individual participants (person-level BIC) and another for the assessment points over the course of trial (assessment-level BIC) (Nagin, 2005).

Additionally, to assess the adequacy of the classification of individuals into trajectory groups, we computed average posterior probability (AvePP) of assignment to each group, odds of correct classification (OCC) based on AvePP, and relative entropy, which is a measure of the degree of classification accuracy of placing participants into a trajectory group based on their posterior probability. These measures also reflect the uncertainty in assignment of individuals to the groups. An AvePP closer to 1, an OCC > 5.0 for all groups, and relative entropy > 0.80 are indicative of high assignment accuracy (Mesidor et al., 2022; Nagin, 2005). The quadratic term, in addition to linear term, for trajectories were included to capture deviations from linearity.

In the second stage of analyses, association of identified groups with baseline sociodemographic characteristics (age, sex, race/ethnicity), characteristics of drug use (use of other substances, injection drug use, lifetime duration of cocaine use), baseline ASI domain scores, and pharmacotherapy arms were assessed using multinominal logistic regression with multi-trajectory groups identified in the previous stage of the analyses as the dependent variable.

Association of trajectory groups with health and psychosocial functioning was assessed in the third stage of analyses, in which change in ASI domain scores from baseline to the last available assessment was the dependent variable and trajectory groups were the independent variables. Linear regression models, adjusted for baseline demographic characteristics (age, sex, race/ethnicity), characteristics of drug use (co-use of other substances, injection drug use, duration of cocaine use), and baseline ASI domain scores were used for these analyses.

GBTM uses maximum likelihood estimation, and as such, the results are robust to data missing completely at random (MCAR) or missing at random (MAR). To assess the sensitivity of the results to data missing not at random (MNAR) and to adjust for such missingness, pattern mixture modeling was used (Enders, 2022). This approach has been used in past GBTM analyses (Dodge et al., 2008). Briefly, as a first step, patterns of missing data for each outcome (i.e., craving, Utox-ascertained cocaine use, and self-reported cocaine use) across assessment points were examined. Next, multi-trajectory modeling (similar to the main analysis) was conducted to identify trajectories of missingness. Lastly, the main multi-trajectory analyses of the study were repeated after including dummy variables for missing data trajectories as covariates in the model.

### 2.4. Sensitivity analyses

Two sets of sensitivity analyses were conducted. In the first set of sensitivity analyses, the multi-trajectory analysis was repeated after limiting the sample to 4 RCTs lasting 12 weeks. In the second set of analyses, the multi-trajectory analyses were limited to the 2 outcome variables of craving and Utox-ascertained cocaine use—similar to our analyses of methamphetamine RCTs (Mojtabai et al., 2024).

Multi-trajectory analyses were conducted using the *traj* plug-in of Stata 18 software (StataCorp LLC, College Station, TX) (Jones and Nagin, 2013). All other analyses were also conducted in Stata 18. Statistical significance was determined based on *p* < .05 (2-tailed).

## 3. Results

### 3.1. Characteristics of participants

Overall, data on 1070 participants from the 6 RCTs were analyzed (n = 610 from 12-week RCTs and n = 460 from 8-week RCTs). Most participants were male (n = 810, 75.7 %) and non-Hispanic Black (n = 637, 59.9 %), followed by non-Hispanic White (n = 296, 27.8 %), Hispanic (n = 109, 10.2 %), and “other” race-ethnicity (n = 22, 2.1 %). The average age of the participants was 41.3 (standard deviation [SD]= 7.9, median=41) years. At baseline, a majority (62.5 %, n = 669) reported having used more than 1 drug in the past 30 days, and 10.0 % (n = 107) had used drugs via injection. On average, participants had used cocaine for 14.1 (SD=7.6, median=14) years. Age, sex and racial/ethnic distribution of participants in each RCT are presented in Supplement C.

The mean numbers of weekly craving assessments (computed as average of up to 3 ratings each week) in 12- and 8-week RCTs were 7.0 (SD=3.5) and 5.2 (SD=2.2), respectively. The respective means were 9.2 (SD=4.0) and 6.7 (SD=2.3) for the number of weekly cocaine use self-reports (based on any report in that week) and 5.3 (SD=5.4) and 6.5 (SD=6.3) for weekly Utox-ascertained cocaine use (based on availability of any assessments in that week).

### 3.2. Trajectories of craving and frequency of cocaine use

**Fig. 1.**
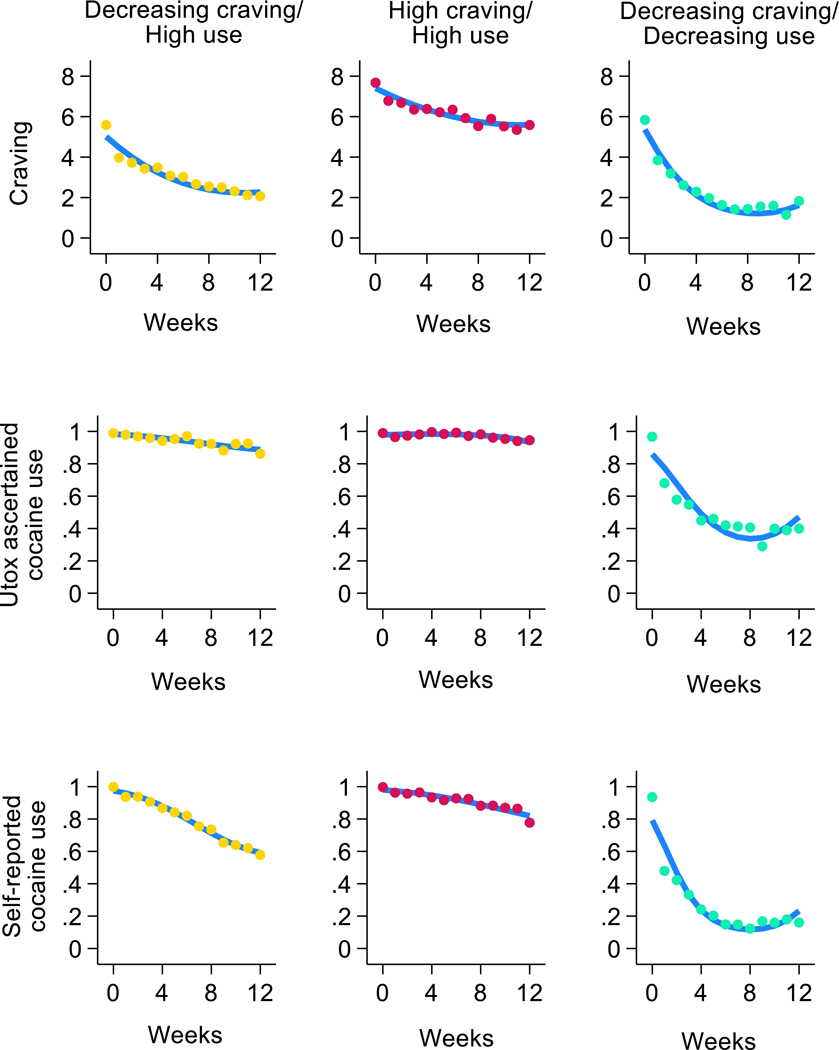
Trajectory groups in multi-trajectory analysis of cocaine craving, urine-toxicology (Utox) ascertained cocaine use and self-reported cocaine use in the 3-group model in randomized controlled trials testing pharmacotherapies in people with cocaine use disorder.

Both craving and cocaine use decreased during the course of RCTs. BSCS ratings decreased from 6.5 (SD=2.4) at baseline to 3.7 (SD=3.0) at the RCTs’ endpoint in the pooled sample. 60.0 % and 83.3 % of the participants used cocaine in their last evaluation based on self-report and Utox, respectively. However, there was significant variability among participants with regard to cocaine craving and use trajectories, as indicated by the large difference between the BIC for the 1-group model vs. models with more than 1 group (Supplement D). Based on considerations of parsimony and comprehensibility of the model (Nagin, 2005), a 3-group model was chosen (Fig. 1). Although the BIC continued to decrease in 4- to 6-group models (Supplement D), the trajectory groups in these larger models were less clearly differentiable and did not represent qualitatively distinct groups (Supplements E-G present trajectory groups for 2- to 5-group models). The 6-group model included a small group with only 5 % of the sample, and the 7-group model did not converge (Supplement D).

The trajectory groups in the 3-group model included a group who experienced marked decreases in craving (from 5.4 [SD=2.2], to 2.4 [SD=2.1]), and in self-reported cocaine use (from 100 % to 61.0 %), but little change in Utox-ascertained cocaine use (from 98.9 % to 88.9 %). This group was labeled Decreasing craving/High use (comprising 36.8 % of participants).

The second group experienced the least amount of change in craving (from 7.7 [SD=1.8] to 5.9 [SD=2.4]), self-reported use (from 99.8 % to 81.9 %), and Utox-ascertained use (from 99.1 % to 95.3 %). This group was labeled the High craving/High use group (comprising 40.0 % of the participants).

Lastly, the third group experienced the largest decrease in craving (from 5.7 [SD=2.7] at baseline to 1.8 [SD=2.4]), self-reported cocaine use (from 92.4 % to 19.2 %), and Utox-ascertained use (from 96.5 % to 52.4 %). This group was labeled Decreasing craving/Decreasing use (comprising 23.2 % of the participants). Most changes in craving and drug use in the Decreasing craving/Decreasing use group occurred in the first 1–2 weeks (Fig. 1). The levels of baseline craving in the Decreasing craving/Decreasing use group and the Decreasing craving/High use group were not significantly different (*t*-test=1.51, df=619, p = 0.131).

### 3.3. Predictors of trajectories

Few of the sociodemographic characteristics were significantly associated with trajectory groups in multinomial logistic regression analysis (Table 1, Supplement H). Higher ASI drug use scores were associated with lower likelihood of being in the Decreasing craving/Decreasing use and Decreasing craving/High use vs. the High craving/High use group (relative risk ratio [RRR]=0.95, 95 % confidence interval [Cl]=0.93–0.97, p < 0.001 and RRR=0.92, 95 % Cl=0.90–0.95, p < 0.001, respectively). Similarly, more years of cocaine use were associated with lower probability of being in the Decreasing craving/Decreasing use group vs. High craving/High use group (RRR=0.96, 95 % CI=0.94–0.99, p = 0.003). In contrast, higher ASI alcohol use scores were associated with higher likelihood of being in the Decreasing craving/High use vs. High craving/High use group (RRR=1.01, 95 % CI=1.005–1.02, p = 0.001), as was use of more than one drug (RRR=1.42, 95 % CI=1.02–1.95, p = 0.035). Additionally, higher ASI employment domain scores were associated with lower likelihood of being in the Decreasing craving/Decreasing use vs. High craving/High use group (RRR=0.99, 95 % CI=0.99–0.999, p = 0.016). Repeating the analyses after excluding the ASI drug domain score did not change the results.

In multinomial logistic regression analyses of medications adjusting for socio-demographic characteristics, drug use and baseline ASI scores, relative to placebo, those on modafinil at 200 mg daily dose (but not other pharmacotherapy) were more likely to be in the Decreasing craving/High use (RRR=5.50, 95 % CI=2.04–14.80, p = 0.001) or Decreasing craving/Decreasing use (RRR=4.84, 95 % CI=1.38–17.00, p = 0.014) groups vs. the High craving/High use group (Table 2).

### 3.4. Association of trajectory groups with changes in ASI domains

**Fig. 2.**
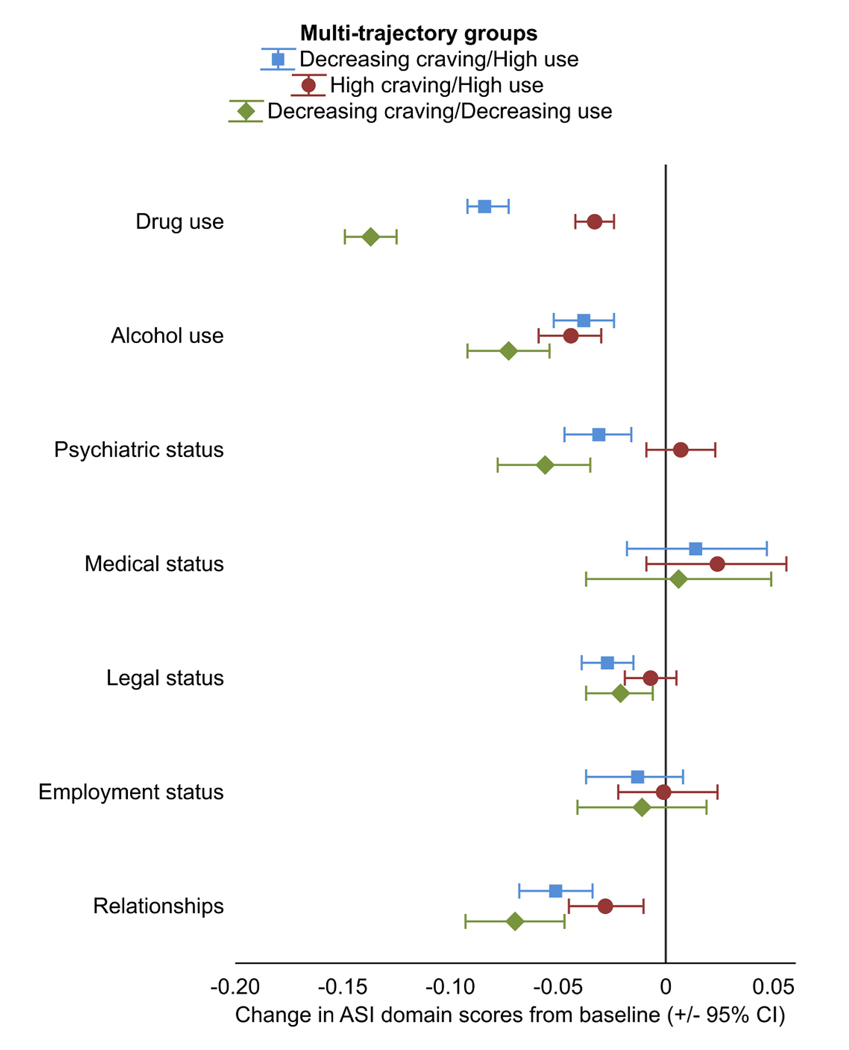
Change in ASI domain scores from baseline to the last assessment in the trajectory groups in randomized controlled trials testing pharmacotherapies in people with cocaine use disorder.

ASI domain scores generally improved from baseline to the last available assessment (Fig. 2; Supplement I). Scores improved significantly in drug and alcohol and relationships domains in all trajectory groups. Additionally, scores on ASI legal and psychiatric domains improved in the Decreasing craving/Decreasing use and Decreasing craving/High use groups. However, the Decreasing craving/Decreasing use group experienced significantly larger improvements in drug use, alcohol use, and relationships domains compared to the High craving/High use group, and in the drug and alcohol use domains compared to Decreasing craving/High use group (Supplement I).

### 3.5. Pattern mixture modeling of missing data

A majority of participants had some missing data on the study outcomes. The pattern of missing data over the 12 weeks is presented in Supplements J-L. A multi-trajectory model with 5 groups had the best fit indices for trajectories of missing data (Supplement M). The missingness trajectories for the 5-group model are presented in Supplement N. Group 1 (comprised of 30.1 % of participants) was characterized by minimal missingness across all variables. Group 3 (14.7 % of participants) was characterized by few missing values on both craving and self-reported use, but substantial missingness for Utox-ascertained use. Groups 2 (24.9 % of participants), 4 (16.6 % of participants) and 5 (13.7 % of participants) were all characterized by increasing trends in missing data for both Utox-ascertained and self-reported cocaine use. However, the onset of missingness varied, occurring later in group 2. These missing data trajectories were significantly associated with outcome trajectories (Supplement O) both in the total sample (Chi-squared=27.36, df=8, p = 0.001) and in analyses limited to 12-week RCTs (Chi-squared=19.60, df=8, p = 0.012). Notably, group 2, characterized by a rapid increase in missing data after week 8, was under-represented in the 12-week RCTs, suggesting that the majority of participants in this trajectory group came from 8-week RCTs (Supplement O).

Repeating the main multi-trajectory analysis with dummy variables for missing data trajectories as covariates did not change the configuration of trajectories in the selected 3-group model (Supplement P). Furthermore, the vast majority of participants in each trajectory group in the main model remained in the same trajectory group in the model adjusting for missing data (Supplement Q).

### 3.6. Sensitivity analyses

Results of the multi-trajectory analyses after excluding 8-week RCTs were similar to the main analyses (Supplement R). Additionally, analyses of predictors of trajectory groups and association with change in ASI were substantively similar to the main analyses (data not shown).

Results of the multi-trajectory analyses limited to craving and Utox-ascertained cocaine use were also consistent with the findings of the main analysis (Supplement S). A model with 3 trajectory groups showed optimal fit to the data. The trajectory groups in this model were similar to those obtained in the main analysis (Supplement T).

## 4. Discussion

There were three main findings in this study. First, the course of cocaine craving and cocaine use during pharmacotherapy RCTs for cocaine use disorder appears to be variable, with reductions occurring for some individuals in both craving and use, whereas others only experiencing reductions in craving and self-reported drug use but not Utox-ascertained use. A third larger group evinced little to no change in either craving or use.

Second, the group who experienced marked reduction in both craving and drug use also reported the largest improvements in outcomes ascertained by ASI, including in psychiatric domain, alcohol use, and relationships. Not surprisingly, more severe drug use and a longer lifetime history of cocaine use at baseline were significantly associated with lower odds of being in the Decreasing craving/Decreasing use group. The Decreasing craving/High use group, in turn, had a higher baseline ASI alcohol use domain score and was more likely to use multiple drugs. These findings suggest that recovery in the course of treatment is multidimensional, and these dimensions are influenced by different predictors. It is also plausible that co-use of alcohol and other drugs diminishes the impact of reduction of craving on cocaine use.

Third, participants who received modafinil 200 mg daily, relative to placebo, were significantly more likely to be in groups experiencing reduction in craving (i.e., Decreasing craving/Decreasing use; Decreasing craving/High use) than in the High craving/High use group. This finding suggests that modafinil at a lower dose may reduce cocaine craving, but not necessarily cocaine use, at least in the context of these RCTs. Modafinil has been shown to be associated with decreased craving for stimulant and other drugs in some trials (Fard et al., 2020; Kampman et al., 2015; Shearer et al., 2009). However, other studies produced negative or inconclusive results on use patterns (Anderson et al., 2012; Lee et al., 2013; Nuijten et al., 2015); for a review, see (Hersey et al., 2021). Our findings echo the results of the analyses reported in the original NIDA-MDS-0004 study (Anderson et al., 2009). In those analyses, the average maximum number of consecutive cocaine non-use days in the modafinil 200 mg arm (12.6 days) was significantly (p = .02) lower than the placebo arm (8.8 days) and change in craving in the modafinil 200 mg arm (p = .04), but not the modafinil 400 mg arm (p = .90), was significantly larger than the placebo arms. Furthermore, the 400 mg dose was associated with a greater number of adverse events and more frequent medication discontinuation (Anderson et al., 2009).

Consistent with the results of our previous study with methamphetamine RCTs (Mojtabai et al., 2024), the trajectory group analysis was robust to missing data and to combining RCTs of different lengths. This finding makes GBTM especially useful for analysis of data from substance use treatment RCTs in which missing data are common.

In interpreting the results of this study, the following limitations should be considered. First, ratings of craving are based on self-report and open to social desirability and/or recall bias. Furthermore, we could not distinguish between tonic craving (a heightened baseline desire for substances) and phasic craving (e.g., cue-induced craving), which may have different trajectories during treatment and different underlying mechanisms (Goodyear and Haass-Koffler, 2020; Hochheimer et al., 2023). Second, the RCTs had relatively short durations. Clinically meaningful changes in cocaine craving and use that were established over many years may require longer treatment. Third, the range of baseline variables included in the RCTs was admittedly limited. A richer set of baseline variables might have revealed other important predictors of these trajectories. In past research, a number of inter-individual genetic and physiological variations have been found to influence craving levels and change in craving in response to treatment (Conrad et al., 2008; Loweth et al., 2014; Terrier et al., 2016; Werner et al., 2019; Xu et al., 2013). Better delineation of these predictors can contribute to the development of personalized treatments for stimulant use disorder.

In conclusion, we were able to examine and define joint trajectories of cocaine craving and cocaine use capitalizing on an unprecedentedly large sample from several RCTs in people with cocaine use disorder. The course of cocaine craving and cocaine use in these RCTs were significantly associated with other measures of recovery, providing good face and concurrent validity for these trajectories. As such, joint trajectories of craving and drug use can serve as meaningful outcomes of cocaine use disorder treatment along with or in place of abstinence which may not be always attainable, especially in the short-term. More prospective and secondary analysis studies are needed to determine whether change in craving may serve as an early indicator of recovery and/or a proxy outcome. Furthermore, the intriguing finding of heterogeneity in the joint trajectories of craving and cocaine use in this study points to inter-individual differences in treatment response, given the possibility that lower craving may or may not be associated with lower use across different people. Exploration of these heterogeneous associations in future research can potentially provide guidance for better matching of treatments to patients based on changes in patterns of craving and drug use early in the course of treatment.

## Supplementary Material

Supplement

## Figures and Tables

**Table 1:** Associations of baseline characteristics with trajectory groups in multinomial logistic regression analyses in randomized controlled trials testing pharmacotherapies in people with cocaine use disorder.

Variable	Decreasing craving/High use vs High craving/High use group			Decreasing craving/Decreasing use vs High craving/High use group		
	Relative risk ratio	95 % CI	*p*	Relative risk ratio	95 % CI	*p*

Sex						
Male	Ref.	–	–	Ref.	–	–
Female	1.10	0.78–1.55	0.598	0.66	0.43–1.02	0.062
Age, years	1.00	0.98–1.02	0.999	0.98	0.95–1.00	0.098
Race/ethnicity						
Non-Hispanic black	Ref.	–	–	Ref.	–	–
Non-Hispanic white	0.80	0.55–1.17	0.254	1.26	0.84–1.91	0.265
Hispanic	0.72	0.42–1.25	0.243	1.10	0.61–1.98	0.756
Other	0.71	0.25–2.05	0.532	0.99	0.31–3.11	0.982
Using > 1 drug (past 30 days)	1.42	1.02–1.95	0.035	1.25	0.86–1.81	0.245
Injection drug use (past 30 days)	0.78	0.47–1.29	0.338	0.87	0.48–1.56	0.510
Years of cocaine use	0.98	0.96–1.00	0.078	0.96	0.94–0.99	0.003
ASI domains						
Drug use	0.95	0.93–0.97	< 0.001	0.92	0.90–0.95	< 0.001
Alcohol use	1.01	1.005–1.02	0.001	1.00	0.99–1.01	0.752
Psychiatric	0.99	0.98–1.00	0.223	1.01	1.00–1.02	0.086
Medical	1.00	0.99–1.00	0.194	1.00	1.00–1.01	0.552
Legal	0.99	0.98–1.00	0.174	0.99	0.98–1.01	0.280
Employment	1.00	0.99–1.00	0.662	0.99	0.99–0.999	0.016
Relationships	0.99	0.99–1.00	0.151	1.00	0.99–1.01	0.584

*Abbreviations*: ASI: Addiction Severity Index; CI: confidence interval.

**Table 2:** Regression results for the association of treatment arms with cocaine craving and use in multi-trajectory group analysis in randomized controlled trials testing pharmacotherapies in people with cocaine use disorder.

Trials and treatment arms^a^	Decreasing craving/High use vs High craving/High use group			Decreasing craving/Decreasing use vs. High craving/High use		
	Relative risk ratio	95 % CI	*p*	Relative risk ratio	95 % CI	*p*

NIDA-CSP−1019 (Elkashef et al., 2006) Placebo	Ref.	–	–	Ref.	–	–
Selegiline transdermal patch (6 mg/day)	1.00	0.61–1.65	0.994	0.47	0.22–1.01	0.052
NIDA-CSP−1021 (Kahn et al., 2009) Placebo	Ref.	–	–	Ref.	–	–
Baclofen (60 mg/day)	0.44	0.18–1.09	0.075	0.62	0.22–1.74	0.368
NIDA-CTO−0007 (Shoptow et al., 2017) Placebo	Ref.	–	–	Ref.	–	–
Cabergoline (0.5 mg/week)	1.27	0.49–3.33	0.623	1.07	0.39–2.90	0.898
NIDA-MDS−0004 (Anderson et al., 2009) Placebo	Ref.	–	–	Ref.	–	–
Modafinil (200 mg/day)	5.50	2.04–14.80	0.001	4.84	1.38–17.00	0.014
Modafinil (400 mg/day)	2.55	0.98–6.62	0.054	1.42	0.46–4.42	0.544
NIDA-CTO−0001 (Winhusen et al., 2007b) Placebo	Ref.	–	–	Ref.	–	–
Reserpine (0.5 mg/day)	0.95	0.37–2.47	0.917	1.00	0.25–4.05	0.995
NIDA-CTO−0012 (Winhusen et al., 2007a) Placebo	Ref.	–	–	Ref.	–	–
Tiagabine 20 mg/day	1.67	0.66–4.25	0.280	1.72	0.63–4.66	0.289

*Abbreviation*: CI: confidence interval.

a. Analyses adjusted for sex, age, race/ethnicity, use of > 1 drugs (past 30 days), injection drug use (past 30 days), years of cocaine use, and baseline ASI domain scores.

## Appendix A. Supporting information

Supplementary data associated with this article can be found in the online version at doi:10.1016/j.drugalcdep.2025.112841.
