# Supplementary material for: Cocaine craving and use during pharmacotherapy trials for cocaine use disorder: A multi-trajectory analysis
Source: Drug Alcohol Depend. Author manuscript; Available in PMC 2026 Jan 27. (PMC12838864; doi:10.1016/j.drugalcdep.2025.112841)
Supplement: Supplement [file NIHMS2131630-supplement-Supplement.pdf]

Cocaine Craving and Use During Pharmacotherapy Trials for Cocaine Use Disorder: A Multi-trajectory Analysis

Supplemental Material

Supplement A: Characteristics of the included randomized controlled trials testing pharmacotherapies in people with cocaine use disorder.

**Study ID**	**Active medication and doses**	**Sample sizes**	**Duration of active phase in weeks**
NIDA-CSP-1019 (Elkashef et al., 2006)	Selegiline transdermal patch (6 mg/day)	Active: 150<br>Placebo: 150	8
NIDA-CSP-1021 (Kahn et al., 2009)	Baclofen (60 mg/day)	Active: 80<br>Placebo: 80	8
NIDA-CTO-0007 (Shoptow et al., 2017)	Cabergoline (0.5 mg/week)	Active: 70<br>Placebo: 70	12
NIDA-MDS-0004 (Anderson et al., 2009)	Modafinil (200 or 400 mg/day)	Modafinil 200 mg/day: 69<br>Modafinil 400 mg/day: 69<br>Placebo: 72	12
NIDA-CTO-0001 (Winhusen et al., 2007b)	Reserpine (0.5 mg/day)	Active: 60<br>Placebo: 59	12
NIDA-CTO-0012 (Winhusen et al., 2007a)	Tiagabine 20 mg/day	Active: 70<br>Placebo: 71	12

Supplement B: Flowchart for selection of randomized controlled trials testing pharmacotherapies in people with cocaine use disorder from the NIDA Data Share.

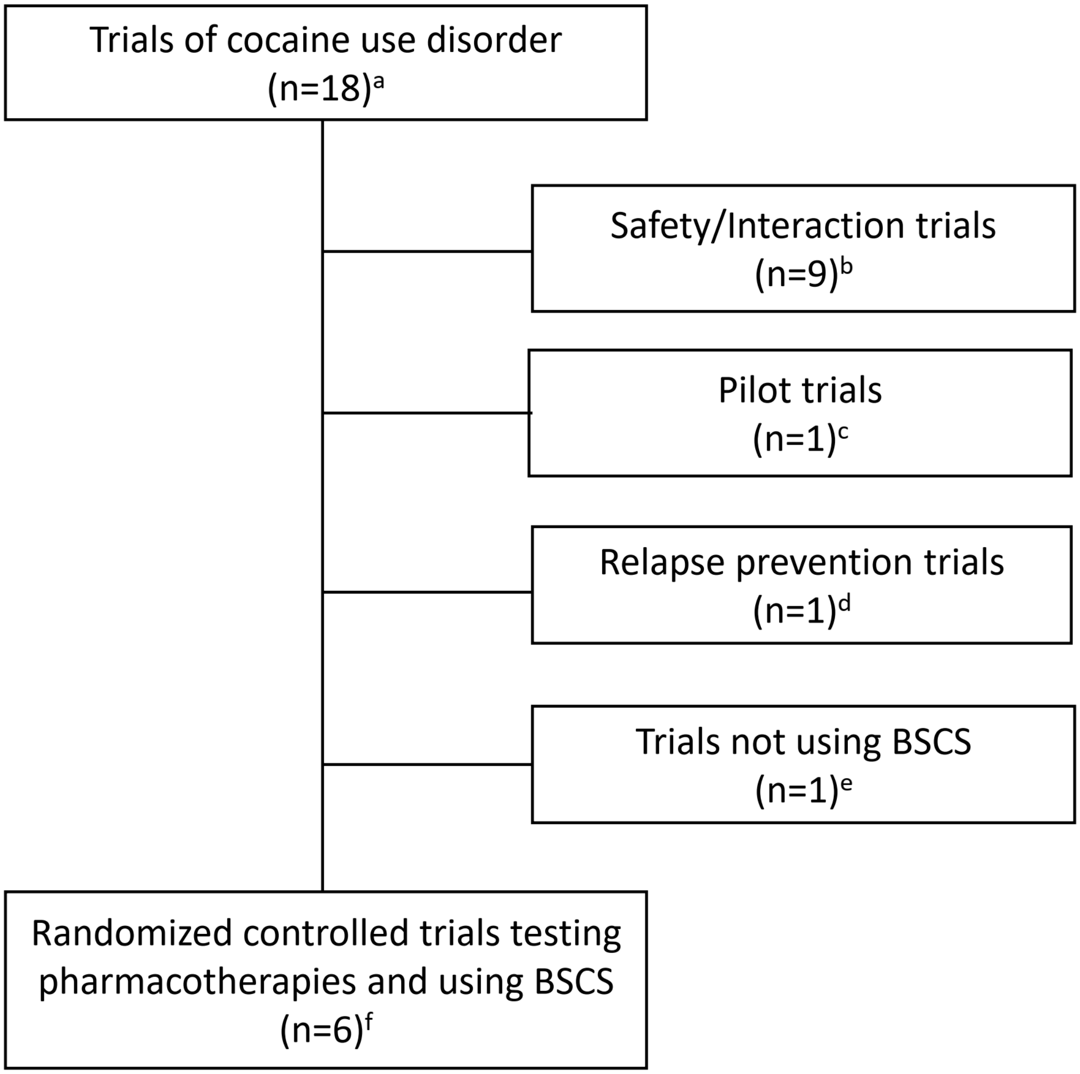

[a] NIDA-CSP-1019, NIDA-CTO-000, NIDA-CTO-0007, NIDA-CTO-0012, NIDA-CSP-1021, NIDA-MDS-0004, NIDA-CPU-0005, NIDA-CPU-0002, NIDA-CTO-0006, NIDA-MDS-0003, NIDA-CPU-0003, NIDA-CPU-0004, NIDA-CPU-0010, NIDA-CTO-0002-, NIDA-CTO-0003, NIDA-CTO-0005, NIDA-CTN-0052, NIDA-CTN-0048

[b] NIDA-CPU-0005, NIDA-CPU-0002, NIDA-CTO-0006, NIDA-MDS-0003, NIDA-CPU-0003, NIDA-CPU-0004, NIDA-CPU-0010, NIDA-CTO-0002-, NIDA-CTO-0003

[c] NIDA-CTO-0005

[d] NIDA-CTN-0052

[e] NIDA-CTN-0048

[f] NIDA-CSP-1019, NIDA-CTO-000, NIDA-CTO-0007, NIDA-CTO-0012, NIDA-CSP-1021, NIDA-MDS-0004

Supplement C: Demographic characteristics of participants in the included randomized controlled trials testing pharmacotherapies in people with cocaine use disorder.

	Age, years		Sex				Race-ethnicity[a]							
			Male		Female		Non-Hispanic White		Non-Hispanic Black		Hispanic		Other	
**Study ID**	**Mean**	**Standard deviation**	**n**	**%**	**n**	**%**	**n**	**%**	**n**	**%**	**n**	**%**	**n**	**%**
NIDA-CSP-1019 (Elkashef et al., 2006)	40.7	7.4	234	78.0	66	22.0	79	26.3	187	62.3	27	9.0	5	1.7
NIDA-CSP-1021 (Kahn et al., 2009)	42.7	8.6	127	79.4	33	20.6	41	25.6	93	58.1	22	13.8	3	1.9
NIDA-CTO-0007 (Shoptow et al., 2017)	40.1	8.2	119	85.0	21	15.0	53	37.9	64	45.7	20	14.3	3	2.1
NIDA-MDS-0004 (Anderson et al., 2009)	41.8	8.3	151	71.9	59	28.1	61	29.1	116	55.2	29	13.8	4	1.9
NIDA-CTO-0001 (Winhusen et al., 2007b)	41.0	7.6	84	70.6	35	29.4	21	17.7	87	73.1	4	3.4	5	4.2
NIDA-CTO-0012 (Winhusen et al., 2007a)	42.0	7.7	95	67.4	46	32.6	41	29.1	90	63.8	7	5.0	2	1.4

a. Data on race-ethnicity were missing in 2 (0.7%) participants in NIDA-CSP-1019, one (0.6%) in NIDA-CSP-1021; 2 (1.7%) in NIDA-CTO-0001, and one (0.7%) in NIDA-CTO-0012.

Supplement D: Model fit indices and other measures of model adequacy for multi-trajectory analysis of cocaine craving, urine-toxicology (Utox) ascertained cocaine use, and self-reported cocaine use in randomized controlled trials testing pharmacotherapies in people with cocaine use disorder.

**Model**	**Estimated group sizes**	**BIC (n=1,019)[a]**	**BIC (n=25,890)[b]**	**AIC**	**Relative entropy**	**AvePP**	**Odds Ratio**
1-group	G1:100%	-25765	-25781	-25740	--	--	--
2-group	G1:65.0%<br>G2:35.0%	-23262	-23296	-23211	0.87	G1:0.96<br>G2:0.96	G1:14.7<br>G2:40.8
3-group	G1:36.8%<br>G2:40.0%<br>G3:23.2%	-22647	-22699	-22569	0.82	G1:0.91<br>G2:0.90<br>G3:0.96	G1:18.0<br>G2:13.4<br>G3:72.4
4-group	G1:20.1%<br>G2:36.6%<br>G3:15.4%<br>G4:27.9%	-22290	-22360	-22184	0.81	G1:0.87<br>G2:0.89<br>G3:0.93<br>G4:0.90	G1:26.6<br>G2:14.4<br>G3:77.2<br>G4:75.7
5-group	G1:19.7%<br>G2:31.3%<br>G3:16.9%<br>G4:18.3%<br>G5:13.8%	-21961	-22048	-21828	0.81	G1:0.90<br>G2:0.85<br>G3:0.88<br>G4:0.86<br>G5:0.91	G1:37.8<br>G2:12.6<br>G3:36.4<br>G4:28.5<br>G5:66.5
6-group	G1:10.8%<br>G2:18.7%<br>G3:20.0%<br>G4:28.6%<br>G5:5.3%<br>G6:16.6%	-21832	-21937	-21672	0.81	G1:0.89<br>G2:0.89<br>G3:0.87<br>G4:0.84<br>G5:0.87<br>G6:0.88	G1:66.7<br>G2:34.3<br>G3:27.2<br>G4:12.7<br>G5:116.4<br>G6:38.0
7-group	--[c]	--[c]	--[c]	--[c]	--[c]	--[c]	--[c]

Abbreviations: BIC: Bayesian Information Criterion; AIC: Akaike Information Criterion; APP: Average Posterior Probability; G1 through G6: trajectory groups 1 through 6.

a. Person-level BIC.

b. Assessment-level BIC.

c. The model did not converge.

Supplement E: Trajectory groups in multi-trajectory analysis of cocaine craving, urine-toxicology (Utox) ascertained cocaine use and self-reported cocaine use in the 2-group model use in randomized controlled trials testing pharmacotherapies in people with cocaine use disorder.

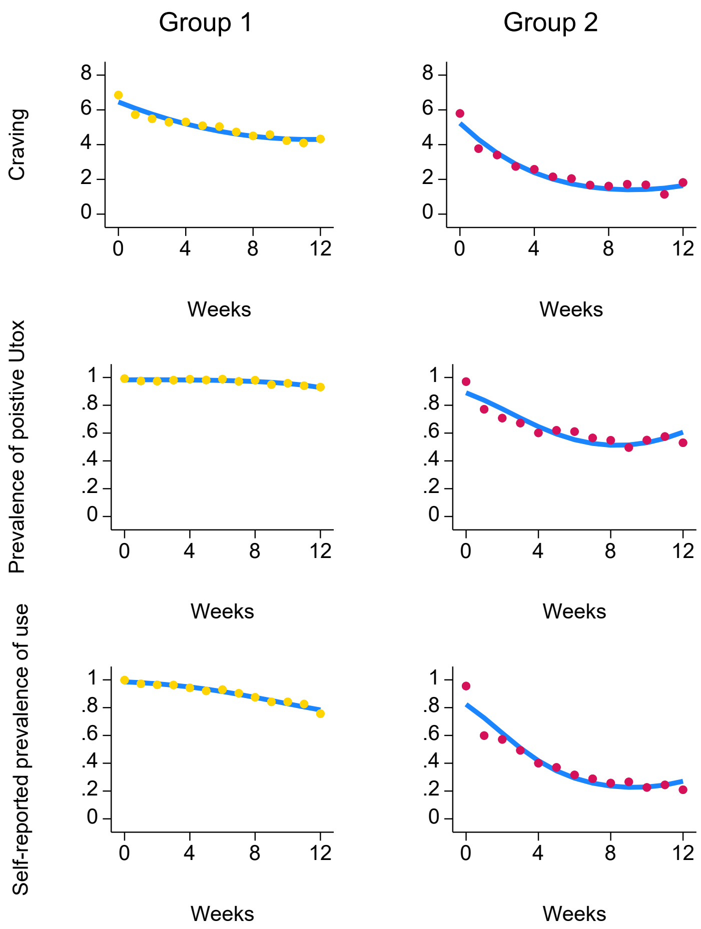

Supplement F: Trajectory groups in multi-trajectory analysis of cocaine craving, urine-toxicology (Utox) ascertained cocaine use and self-reported cocaine use in the 4-group model in randomized controlled trials testing pharmacotherapies in people with cocaine use disorder.

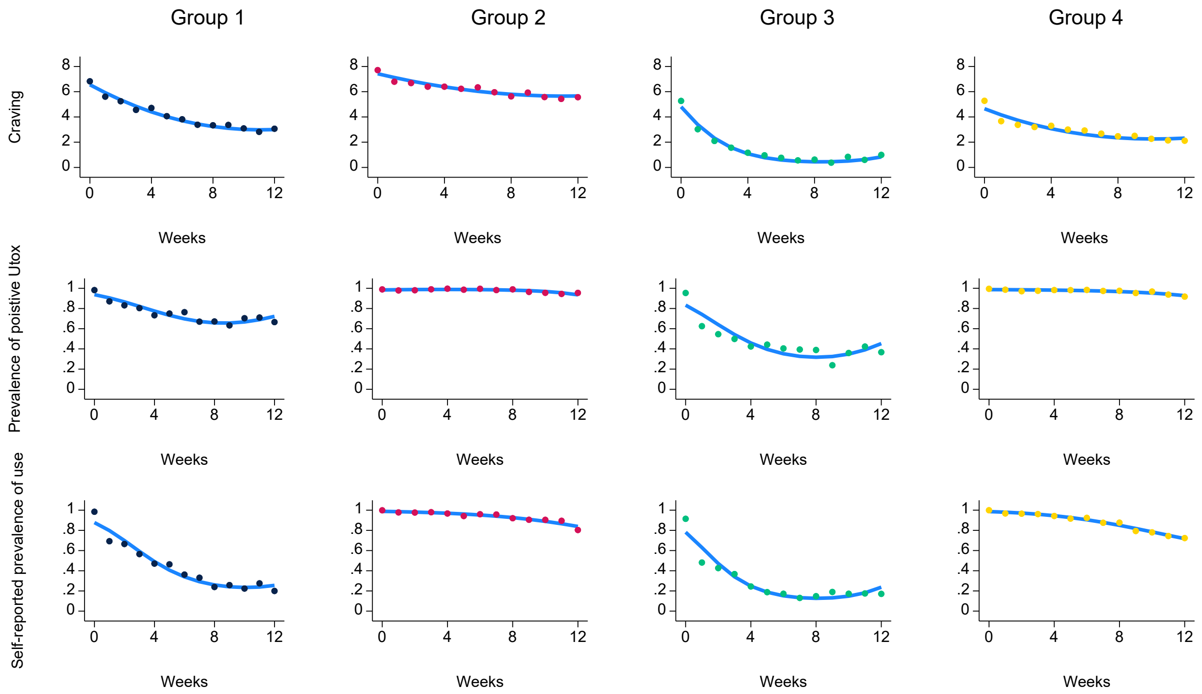

Supplement G: Trajectory groups in multi-trajectory analysis of cocaine craving, urine-toxicology (Utox) ascertained cocaine use and self-reported cocaine use in the 5-group model in randomized controlled trials testing pharmacotherapies in people with cocaine use disorder.

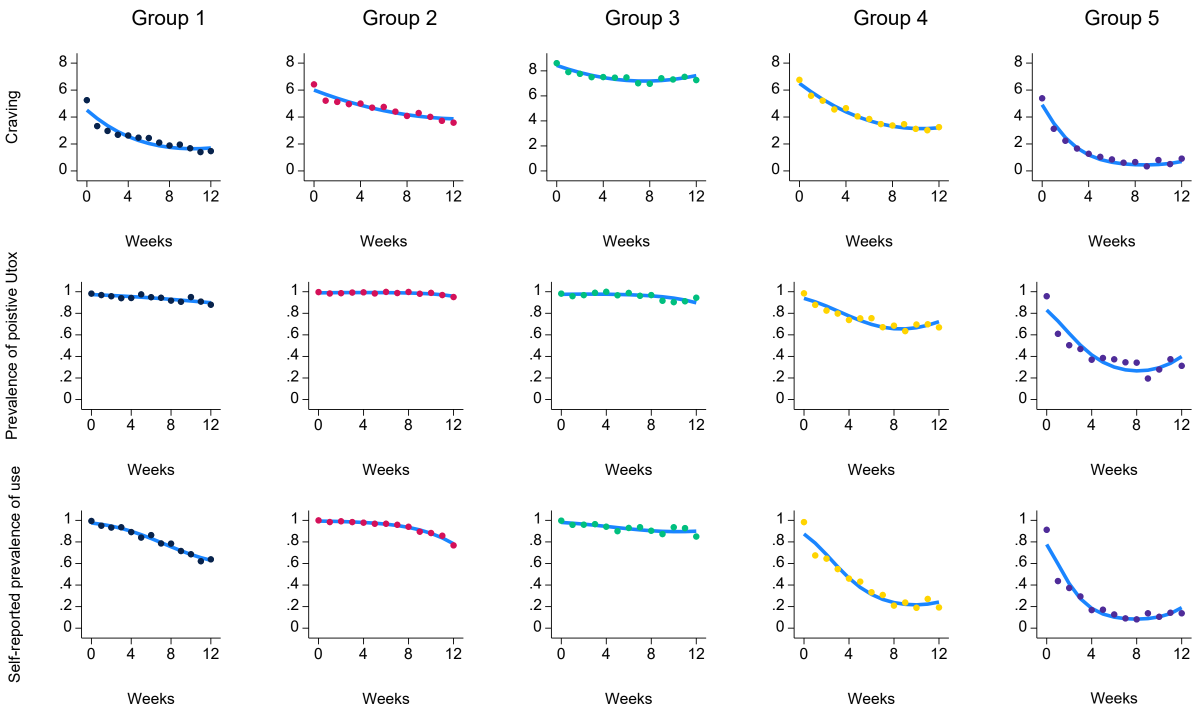

Supplement H: Baseline characteristics of participants in randomized controlled trials testing pharmacotherapies in people with cocaine use disorder based on assignment to groups in multi-trajectory analysis in randomized controlled trials testing pharmacotherapies in people with cocaine use disorder.

Variable	Multi-trajectory groups					
	**Decreasing craving/High use**		**High craving/High use**		**Decreasing craving /Decreasing use**	
	**n**	**%**	**n**	**%**	**n**	**%**
Sex						
Male	275	73.9	328	73.2	207	82.8
Female	97	26.1	120	26.8	43	17.2
Race/ethnicity						
Non-Hispanic black	248	67.2	266	59.4	123	49.8
Non-Hispanic white	86	23.3	122	27.2	88	35.6
Hispanic	29	7.9	49	10.9	31	12.6
Other	6	1.6	11	2.5	5	2.0
Using > 1 drug (past 30 days)	237	63.7	277	61.8	155	62.0
Injection drug use (past 30 days)	32	8.6	55	12.3	20	8.0
	**Mean**	**SD**	**Mean**	**SD**	**Mean**	**SD**
Age, years	41.7	7.1	42.0	8.3	39.6	8.4
Years of cocaine use	13.8	7.1	15.2	7.9	12.4	7.6
ASI domains						
Drug use	0.24	0.09	0.27	0.08	0.22	0.08
Alcohol use	0.22	0.22	0.20	0.20	0.18	0.19
Psychiatric status	0.10	0.16	0.14	0.18	0.14	0.17
Medical status	0.14	0.26	0.19	0.29	0.16	0.28
Legal status	0.06	0.13	0.08	0.16	0.06	0.14
Employment status	0.54	0.34	0.53	0.32	0.45	0.31
Relationships	0.16	0.18	0.21	0.21	0.18	0.18

Abbreviations: ASI: Addiction Severity Index; SD: Standard Deviation.

Supplement I: Change in Addiction Severity Index (ASI) domain scores from baseline to the last available week according to multi-trajectory groups in randomized controlled trials testing pharmacotherapies in people with cocaine use disorder.[a]

ASI domains	Decreasing craving/ High use (DH)			High craving/ High use (HH)			Decreasing craving/ Decreasing use (DD)			Group comparisons
	M	95% CI	*P*	M	95% CI	*P*	M	95% CI	*p*	
Drug use	-0.08	-0.09, -0.08	<0.001	-0.03	-0.04, -0.02	<0.001	-0.14	-0.15, -0.13	<0.001	DD>DH>HH
Alcohol use	-0.04	-0.05, -0.02	<0.001	-0.04	-0.06, -0.03	<0.001	-0.07	-0.09, -0.05	<0.001	DD>HH, DD>DH
Psychiatric status	-0.03	-0.05, -0.02	<0.001	0.01	-0.01, 0.02	0.375	-0.06	-0.08, -0.04	<0.001	DD>HH, DH>HH
Medical status	0.01	-0.02, 0.05	0.394	0.02	-0.01, 0.06	0.149	0.01	-0.04, 0.05	0.773	--
Legal status	-0.03	-0.04, -0.02	<0.001	-0.01	-0.02, 0.01	0.247	-0.02	-0.04, -0.01	0.007	DH>HH
Employment status	-0.01	-0.04, 0.01	0.255	-0.00	-0.02, 0.02	0.936	-0.01	-0.04, 0.02	0.458	--
Relationships	-0.05	-0.07, -0.03	<0.001	-0.03	-0.05, -0.01	0.002	-0.07	-0.09, -0.05	<0.001	DD>HH

Abbreviations: M: Mean change from baseline to the last available assessment week, CI: Confidence Interval.

[a.] Analyses adjusted for sex, age, race/ethnicity, use of >1 drugs (past 30 days), injection drug use (past 30 days), years of cocaine use, and baseline ASI domain scores.

Supplement J: Patterns of missing responses to craving questionnaire from week 1 to week 12 (a value of 0 indicates missing response in that week) in randomized controlled trials testing pharmacotherapies in people with cocaine use disorder.

	Weeks											
Percent	1	2	3	4	5	6	7	8	9	10	11	12
1%	1	1	1	1	1	1	1	1	1	1	1	1
Missing 1 week:												
4	1	1	1	1	1	1	1	1	1	1	1	0
1	1	1	1	1	1	1	1	1	1	1	0	1
<1	1	1	1	1	1	1	1	1	1	0	1	1
<1	1	1	1	1	1	1	0	1	1	1	1	1
<1	1	1	1	1	1	0	1	1	1	1	1	1
<1	1	1	1	1	0	1	1	1	1	1	1	1
<1	1	1	1	0	1	1	1	1	1	1	1	1
<1	1	1	0	1	1	1	1	1	1	1	1	1
<1	1	0	1	1	1	1	1	1	1	1	1	1
Missing 2 weeks:												
<1	1	1	1	1	1	1	1	1	1	0	1	0
<1	1	1	1	1	1	1	1	1	1	0	0	1
<1	1	1	1	1	1	1	1	1	0	1	1	0
<1	1	1	1	1	1	1	1	1	0	1	0	1
<1	1	1	1	1	1	1	1	0	1	1	1	0
<1	1	1	1	1	1	1	1	0	1	1	0	1
<1	1	1	1	1	1	1	0	1	1	1	1	0
<1	1	1	1	1	1	1	0	1	1	1	0	1
<1	1	1	1	1	1	1	0	1	1	0	1	1
<1	1	1	1	1	1	0	1	1	1	1	1	0
<1	1	1	1	1	1	0	1	1	1	1	0	1
<1	1	1	1	1	1	0	1	1	1	0	1	1
<1	1	1	1	1	1	0	1	1	0	1	1	1
<1	1	1	1	1	1	0	1	0	1	1	1	1
<1	1	1	1	1	1	0	0	1	1	1	1	1
<1	1	1	1	1	0	1	1	1	1	1	1	0
<1	1	1	1	1	0	1	1	1	1	1	0	1
<1	1	1	1	1	0	1	1	1	1	0	1	1
<1	1	1	1	0	1	1	1	1	1	1	1	0
<1	1	1	1	0	1	1	1	1	1	1	0	1
<1	1	1	1	0	1	1	1	1	1	0	1	1
<1	1	1	1	0	0	1	1	1	1	1	1	1
<1	1	1	0	1	1	1	1	1	1	1	1	0
<1	1	1	0	1	1	1	1	1	1	1	0	1
<1	1	1	0	1	1	1	1	1	1	0	1	1
<1	1	1	0	1	0	1	1	1	1	1	1	1
<1	1	0	1	1	1	1	1	1	1	1	1	0
<1	1	0	1	1	1	1	1	1	1	1	0	1
<1	1	0	1	1	1	1	1	1	1	0	1	1
<1	1	0	1	1	1	1	1	1	0	1	1	1

```
     <1      |  1  0  1  1   1  1  0  1   1  1  1  1
     <1      |  1  0  1  1   1  0  1  1   1  1  1  1
     <1      |  1  0  0  1   1  1  1  1   1  1  1  1
     <1      |  0  1  1  1   1  1  1  1   1  1  1  0
     <1      |  0  1  1  1   1  1  1  1   1  1  0  1
     <1      |  0  1  1  1   0  1  1  1   1  1  1  1
Missing 3 weeks:
     <1      |  1  1  1  1   1  1  1  1   1  0  0  0
     <1      |  1  1  1  1   1  1  1  0   1  0  1  0
     <1      |  1  1  1  1   1  1  0  1   1  0  1  0
     <1      |  1  1  1  1   1  1  0  1   1  0  0  1
     <1      |  1  1  1  1   1  1  0  1   0  1  1  0
     <1      |  1  1  1  1   1  1  0  1   0  1  0  1
     <1      |  1  1  1  1   1  1  0  0   1  1  0  1
     <1      |  1  1  1  1   1  0  1  1   1  0  1  0
     <1      |  1  1  1  1   1  0  1  1   0  1  1  0
     <1      |  1  1  1  1   1  0  1  1   0  1  0  1
     <1      |  1  1  1  1   1  0  1  0   1  1  1  0
     <1      |  1  1  1  1   1  0  1  0   0  1  1  1
     <1      |  1  1  1  1   1  0  0  1   1  1  1  0
     <1      |  1  1  1  1   1  0  0  1   1  0  1  1
     <1      |  1  1  1  1   1  0  0  1   0  1  1  1
     <1      |  1  1  1  1   0  1  1  1   1  0  1  0
     <1      |  1  1  1  1   0  1  1  1   0  1  1  0
     <1      |  1  1  1  1   0  1  1  1   0  1  0  1
     <1      |  1  1  1  1   0  0  1  1   1  1  1  0
     <1      |  1  1  1  1   0  0  1  1   1  1  0  1
     <1      |  1  1  1  0   1  1  1  1   0  1  1  0
     <1      |  1  1  1  0   1  1  1  1   0  1  0  1
     <1      |  1  1  1  0   1  1  1  1   0  0  1  1
     <1      |  1  1  1  0   1  1  1  0   1  1  1  0
     <1      |  1  1  1  0   1  1  1  0   1  1  0  1
     <1      |  1  1  1  0   1  1  0  1   1  1  1  0
     <1      |  1  1  1  0   1  1  0  1   1  1  0  1
     <1      |  1  1  1  0   0  1  1  1   1  1  0  1
     <1      |  1  1  0  1   1  1  1  1   1  0  1  0
     <1      |  1  1  0  1   1  1  1  1   0  1  1  0
     <1      |  1  1  0  1   1  1  1  0   1  1  1  0
     <1      |  1  1  0  1   1  1  1  0   1  1  0  1
     <1      |  1  1  0  1   1  1  1  0   1  0  1  1
     <1      |  1  1  0  1   1  1  0  1   1  1  1  0
     <1      |  1  1  0  1   1  1  0  1   1  1  0  1
     <1      |  1  1  0  1   1  0  1  1   1  1  1  0
     <1      |  1  1  0  1   1  0  1  1   1  1  0  1
     <1      |  1  1  0  1   0  1  1  1   1  1  1  0
     <1      |  1  1  0  1   0  1  1  1   1  1  0  1
     <1      |  1  1  0  1   0  1  1  1   1  0  1  1
     <1      |  1  0  1  1   1  1  1  1   1  0  1  0
     <1      |  1  0  1  1   1  1  1  0   1  1  1  0
     <1      |  1  0  1  1   1  1  0  1   1  1  1  0
     <1      |  1  0  1  1   1  1  0  1   1  1  0  1
     <1      |  1  0  1  1   1  0  1  1   0  1  1  1
```

<1	1	0	1	1	0	1	1	1	1	1	1	0
<1	1	0	1	0	1	1	1	1	1	1	1	0
<1	1	0	0	1	1	1	1	1	1	1	1	0
<1	1	0	0	1	1	1	1	1	1	1	0	1
<1	0	1	1	1	1	1	1	1	1	0	1	0
<1	0	1	1	1	1	1	1	1	0	1	1	0
<1	0	1	1	1	1	1	1	1	0	0	1	1
<1	0	1	1	1	1	1	1	0	1	1	1	0
<1	0	1	1	1	1	1	0	1	1	1	1	0
<1	0	1	1	1	1	1	0	1	1	0	1	1
<1	0	1	1	1	1	0	1	1	1	1	1	0
<1	0	1	1	1	1	0	1	0	1	1	1	1
<1	0	1	1	1	0	1	1	1	1	1	1	0
<1	0	1	1	1	0	1	1	1	1	1	0	1
<1	0	1	1	0	1	1	1	1	1	1	1	0
<1	0	0	1	1	1	0	1	1	1	1	1	1
**Missing 4 weeks:**												
5	1	1	1	1	1	1	1	1	0	0	0	0
3	1	1	1	1	1	1	1	0	1	0	0	0
<1	1	1	1	1	1	1	1	0	0	0	1	0
<1	1	1	1	1	1	1	0	1	1	0	0	0
<1	1	1	1	1	1	0	1	1	0	0	1	0
<1	1	1	1	1	1	0	1	1	0	0	0	1
<1	1	1	1	1	1	0	1	0	0	1	1	0
<1	1	1	1	1	0	1	1	1	1	0	0	0
<1	1	1	1	1	0	1	1	1	0	0	1	0
<1	1	1	1	1	0	0	1	0	1	1	1	0
<1	1	1	1	0	1	1	1	0	1	1	0	0
<1	1	1	1	0	1	1	1	0	1	0	1	0
<1	1	1	1	0	1	1	1	0	1	0	0	1
<1	1	1	1	0	1	0	1	1	1	0	1	0
<1	1	1	1	0	0	1	1	1	1	0	1	0
<1	1	1	1	0	0	1	1	1	0	1	1	0
<1	1	1	1	0	0	1	1	0	1	1	1	0
<1	1	1	0	1	1	1	1	1	0	1	0	0
<1	1	1	0	1	1	1	1	0	1	0	1	0
<1	1	1	0	1	1	1	1	0	0	1	0	1
<1	1	1	0	1	1	1	0	1	0	1	1	0
<1	1	1	0	1	1	0	1	1	1	0	1	0
<1	1	1	0	1	1	0	1	1	0	1	0	1
<1	1	1	0	1	1	0	1	0	1	1	0	1
<1	1	1	0	1	1	0	0	1	1	1	1	0
<1	1	1	0	1	1	0	0	1	1	1	0	1
<1	1	1	0	1	0	1	1	1	1	0	0	1
<1	1	1	0	1	0	1	1	0	1	1	0	1
<1	1	1	0	1	0	0	1	1	1	1	0	1
<1	1	1	0	0	1	1	1	1	0	1	1	0
<1	1	0	1	1	1	1	1	1	1	0	0	0
<1	1	0	1	1	1	1	1	1	0	1	0	0
<1	1	0	1	1	1	1	1	0	1	1	0	0
<1	1	0	1	1	1	1	1	0	1	0	1	0
<1	1	0	1	1	1	1	1	0	1	0	0	1

<1	|	1	0	1	1	1	1	0	1	1	0	1	0
<1	|	1	0	1	1	1	1	0	1	0	1	1	0
<1	|	1	0	1	1	1	0	1	1	1	0	1	0
<1	|	1	0	1	1	1	0	1	1	0	1	1	0
<1	|	1	0	1	1	1	0	0	1	1	1	1	0
<1	|	1	0	1	1	1	0	0	1	1	1	0	1
<1	|	1	0	1	1	1	0	0	1	0	1	1	1
<1	|	1	0	1	1	0	1	1	1	1	0	1	0
<1	|	1	0	1	0	1	1	1	1	0	1	1	0
<1	|	1	0	1	0	0	1	1	1	1	1	0	1
<1	|	1	0	1	0	0	1	1	1	1	0	1	1
<1	|	1	0	1	0	0	1	1	1	0	1	1	1
<1	|	1	0	0	1	1	1	1	1	1	0	1	0
<1	|	1	0	0	1	1	1	1	0	1	1	1	0
<1	|	1	0	0	1	1	1	0	1	1	1	1	0
<1	|	1	0	0	1	1	1	0	1	1	1	0	1
<1	|	1	0	0	1	0	1	1	1	1	1	1	0
<1	|	1	0	0	1	0	1	1	1	1	1	0	1
<1	|	0	1	1	1	1	1	1	1	0	0	1	0
<1	|	0	1	1	1	1	1	1	0	1	0	1	0
<1	|	0	1	1	1	1	1	1	0	1	0	0	1
<1	|	0	1	1	1	1	1	0	1	1	1	0	0
<1	|	0	1	1	1	1	1	0	1	1	0	0	1
<1	|	0	1	1	1	1	0	1	1	1	0	1	0
<1	|	0	1	1	1	1	0	0	1	1	1	1	0
<1	|	0	1	1	1	1	0	0	1	1	0	1	1
<1	|	0	1	1	1	1	0	0	1	0	1	1	1
<1	|	0	1	1	1	0	1	1	1	0	1	1	0
<1	|	0	1	1	1	0	1	1	0	1	0	1	1
<1	|	0	1	1	0	1	1	1	0	1	1	1	0
<1	|	0	1	1	0	0	1	1	1	1	1	1	0
<1	|	0	1	0	1	1	1	1	1	1	1	0	0
**Missing 5 weeks:**													
<1	|	1	1	1	1	1	1	1	0	0	0	0	0
1	|	1	1	1	1	1	1	0	1	0	0	0	0
<1	|	1	1	1	1	1	1	0	0	1	0	0	0
1	|	1	1	1	1	1	0	1	1	0	0	0	0
<1	|	1	1	1	1	1	0	1	0	1	0	0	0
<1	|	1	1	1	1	1	0	0	1	0	1	0	0
<1	|	1	1	1	1	0	1	1	1	0	0	0	0
<1	|	1	1	1	1	0	1	1	0	1	0	0	0
<1	|	1	1	1	1	0	1	1	0	0	1	0	0
1	|	1	1	1	0	1	1	1	1	0	0	0	0
<1	|	1	1	1	0	1	1	1	0	1	0	0	0
<1	|	1	1	1	0	1	1	1	0	0	1	0	0
<1	|	1	1	1	0	1	1	0	1	1	0	0	0
<1	|	1	1	1	0	0	1	1	1	1	0	0	0
<1	|	1	1	1	0	0	1	1	1	0	1	0	0
<1	|	1	1	1	0	0	1	1	0	1	1	0	0
<1	|	1	1	1	0	0	1	0	1	1	0	1	0
<1	|	1	1	1	0	0	1	0	1	0	1	0	1
<1	|	1	1	1	0	0	0	1	1	1	1	0	0

```
     <1     |  1  1  0  1    1  1  1  1    0  0  0  0
     <1     |  1  1  0  1    1  1  1  0    1  0  0  0
     <1     |  1  1  0  1    1  1  1  0    0  1  0  0
     <1     |  1  1  0  1    1  1  0  1    1  0  0  0
     <1     |  1  1  0  1    1  0  1  1    1  0  0  0
     <1     |  1  1  0  1    1  0  1  0    1  1  0  0
     <1     |  1  1  0  1    0  1  1  1    1  0  0  0
     <1     |  1  1  0  1    0  1  0  1    0  1  0  1
     <1     |  1  0  1  1    1  1  1  1    0  0  0  0
     <1     |  1  0  1  1    1  1  1  0    1  0  0  0
     <1     |  1  0  1  1    1  1  1  0    0  1  0  0
     <1     |  1  0  1  1    1  0  1  1    1  0  0  0
     <1     |  1  0  1  1    1  0  1  0    0  1  0  1
     <1     |  1  0  1  1    0  1  1  1    1  0  0  0
     <1     |  1  0  1  0    1  1  1  1    1  0  0  0
     <1     |  1  0  0  1    1  1  1  0    1  0  1  0
     <1     |  1  0  0  1    1  1  0  1    0  1  1  0
     <1     |  1  0  0  1    1  1  0  1    0  1  0  1
     <1     |  1  0  0  1    1  0  1  1    1  0  1  0
     <1     |  1  0  0  1    0  1  1  1    1  0  0  1
     <1     |  0  1  1  1    1  1  1  1    0  0  0  0
     <1     |  0  1  1  1    1  1  1  0    1  0  0  0
     <1     |  0  1  1  1    1  1  1  0    0  1  0  0
     <1     |  0  1  1  1    1  1  0  1    1  0  0  0
     <1     |  0  1  1  1    1  1  0  1    0  1  0  0
     <1     |  0  1  1  1    1  1  0  0    1  1  0  0
     <1     |  0  1  1  1    1  0  1  1    1  0  0  0
     <1     |  0  1  1  1    1  0  1  1    0  1  0  0
     <1     |  0  1  1  1    1  0  0  1    1  0  1  0
     <1     |  0  1  1  1    0  1  1  1    1  0  0  0
     <1     |  0  1  1  1    0  1  1  1    0  1  0  0
     <1     |  0  1  1  0    1  1  1  0    0  1  1  0
     <1     |  0  1  1  0    0  1  1  1    0  1  1  0
     <1     |  0  1  1  0    0  1  1  1    0  1  0  1
     <1     |  0  0  1  0    1  1  1  1    1  0  1  0
Missing 6 weeks:
     <1     |  1  1  1  1    1  1  0  0    0  0  0  0
     <1     |  1  1  1  1    1  0  1  0    0  0  0  0
     <1     |  1  1  1  1    1  0  0  1    0  0  0  0
     <1     |  1  1  1  1    1  0  0  0    1  0  0  0
     <1     |  1  1  1  1    1  0  0  0    0  1  0  0
     <1     |  1  1  1  1    1  0  0  0    0  0  1  0
     <1     |  1  1  1  1    0  1  1  0    0  0  0  0
     <1     |  1  1  1  1    0  1  0  0    0  0  1  0
     <1     |  1  1  1  1    0  0  1  1    0  0  0  0
     <1     |  1  1  1  0    1  1  1  0    0  0  0  0
     <1     |  1  1  1  0    1  1  0  1    0  0  0  0
     <1     |  1  1  1  0    1  1  0  0    1  0  0  0
     <1     |  1  1  1  0    0  1  1  1    0  0  0  0
     <1     |  1  1  1  0    0  1  1  0    1  0  0  0
     <1     |  1  1  1  0    0  1  1  0    0  1  0  0
     <1     |  1  1  0  1    1  1  1  0    0  0  0  0
```

<1	1	1	0	1	1	1	0	1	0	0	0	0
<1	1	1	0	1	1	1	0	0	1	0	0	0
<1	1	1	0	1	1	0	1	1	0	0	0	0
<1	1	1	0	1	1	0	1	0	1	0	0	0
<1	1	1	0	1	0	1	1	1	0	0	0	0
<1	1	1	0	1	0	1	1	0	1	0	0	0
<1	1	0	1	1	1	1	1	0	0	0	0	0
<1	1	0	1	1	1	1	0	1	0	0	0	0
<1	1	0	1	1	1	0	1	1	0	0	0	0
<1	1	0	1	1	1	0	1	0	1	0	0	0
<1	1	0	1	1	0	1	1	1	0	0	0	0
<1	1	0	1	1	0	1	1	0	1	0	0	0
<1	1	0	1	0	1	1	1	1	0	0	0	0
<1	1	0	1	0	1	1	1	0	1	0	0	0
<1	1	0	1	0	1	1	0	1	1	0	0	0
<1	1	0	1	0	1	1	0	1	0	0	0	1
<1	1	0	1	0	1	0	0	1	1	1	0	0
<1	1	0	1	0	1	0	0	0	1	1	1	0
<1	1	0	1	0	0	1	0	0	1	0	1	1
<1	1	0	0	1	1	1	1	1	0	0	0	0
<1	1	0	0	1	1	1	1	0	1	0	0	0
<1	1	0	0	1	1	0	1	1	1	0	0	0
<1	1	0	0	1	1	0	1	1	0	0	1	0
<1	0	1	1	1	1	1	1	0	0	0	0	0
<1	0	1	1	1	1	1	0	1	0	0	0	0
<1	0	1	1	1	1	0	1	1	0	0	0	0
<1	0	1	1	1	1	0	1	0	1	0	0	0
<1	0	1	1	1	0	1	1	1	0	0	0	0
<1	0	1	1	1	0	1	1	0	1	0	0	0
<1	0	1	1	1	0	0	0	1	0	1	1	0
<1	0	1	1	0	1	1	1	1	0	0	0	0
<1	0	1	1	0	1	1	1	0	1	0	0	0
<1	0	1	0	1	1	1	0	1	1	0	0	0
<1	0	0	1	1	1	1	1	1	0	0	0	0

Missing 7 weeks:

<1	1	1	1	1	1	0	0	0	0	0	0	0
<1	1	1	1	1	0	1	0	0	0	0	0	0
<1	1	1	1	1	0	0	1	0	0	0	0	0
<1	1	1	1	1	0	0	0	1	0	0	0	0
<1	1	1	1	1	0	0	0	0	1	0	0	0
<1	1	1	1	0	1	1	0	0	0	0	0	0
<1	1	1	1	0	0	1	1	0	0	0	0	0
<1	1	1	1	0	0	1	0	1	0	0	0	0
<1	1	1	1	0	0	1	0	0	0	0	1	0
<1	1	1	1	0	0	0	0	0	1	0	1	0
<1	1	1	1	0	0	0	0	0	0	1	0	1
<1	1	1	0	1	1	1	0	0	0	0	0	0
<1	1	1	0	1	1	0	1	0	0	0	0	0
<1	1	1	0	1	1	0	0	0	0	0	1	0
<1	1	1	0	1	1	0	0	0	0	0	0	1
<1	1	1	0	1	0	1	0	1	0	0	0	0
<1	1	1	0	0	1	1	0	1	0	0	0	0

<1	1	1	0	0	1	0	1	1	0	0	0	0
<1	1	1	0	0	0	1	1	1	0	0	0	0
<1	1	0	1	1	1	1	0	0	0	0	0	0
<1	1	0	1	1	1	0	1	0	0	0	0	0
<1	1	0	1	1	1	0	0	1	0	0	0	0
<1	1	0	1	1	1	0	0	0	0	0	0	1
<1	1	0	1	1	0	0	1	1	0	0	0	0
<1	1	0	1	0	1	1	1	0	0	0	0	0
<1	1	0	1	0	1	1	0	1	0	0	0	0
<1	1	0	1	0	1	0	1	1	0	0	0	0
<1	1	0	1	0	0	1	1	1	0	0	0	0
<1	1	0	1	0	0	0	0	1	0	1	0	1
<1	1	0	0	1	1	1	1	0	0	0	0	0
<1	1	0	0	1	1	0	1	0	1	0	0	0
<1	1	0	0	1	1	0	0	1	1	0	0	0
<1	1	0	0	1	0	1	1	1	0	0	0	0
<1	1	0	0	1	0	1	1	0	1	0	0	0
<1	1	0	0	0	1	1	0	0	0	1	1	0
<1	0	1	1	1	1	1	0	0	0	0	0	0
<1	0	1	1	1	1	0	1	0	0	0	0	0
<1	0	1	1	1	1	0	0	1	0	0	0	0
<1	0	1	1	1	0	1	1	0	0	0	0	0
<1	0	1	1	1	0	1	0	1	0	0	0	0
<1	0	1	1	1	0	1	0	0	1	0	0	0
<1	0	1	1	1	0	0	1	1	0	0	0	0
<1	0	1	1	0	1	1	0	1	0	0	0	0
<1	0	1	1	0	0	1	1	1	0	0	0	0
<1	0	1	1	0	0	1	1	0	1	0	0	0
<1	0	1	0	1	1	1	0	1	0	0	0	0
<1	0	1	0	1	1	1	0	0	1	0	0	0
<1	0	1	0	0	1	1	1	0	1	0	0	0
<1	0	0	0	0	1	0	0	1	1	1	1	0
**Missing 8 weeks:**												
1	1	1	1	1	0	0	0	0	0	0	0	0
<1	1	1	1	0	0	1	0	0	0	0	0	0
<1	1	1	0	1	0	1	0	0	0	0	0	0
<1	1	1	0	1	0	0	1	0	0	0	0	0
<1	1	0	1	1	1	0	0	0	0	0	0	0
<1	1	0	1	1	0	1	0	0	0	0	0	0
<1	1	0	1	0	0	1	1	0	0	0	0	0
<1	1	0	1	0	0	1	0	0	0	1	0	0
<1	1	0	1	0	0	0	1	0	1	0	0	0
<1	1	0	1	0	0	0	0	1	1	0	0	0
<1	1	0	0	1	1	1	0	0	0	0	0	0
<1	1	0	0	1	0	1	1	0	0	0	0	0
<1	1	0	0	1	0	1	0	1	0	0	0	0
<1	1	0	0	1	0	1	0	0	0	1	0	0
<1	1	0	0	0	0	1	0	0	0	1	0	1
<1	0	1	1	1	1	0	0	0	0	0	0	0
<1	0	1	1	1	0	1	0	0	0	0	0	0
<1	0	1	1	1	0	0	0	0	0	1	0	0
<1	0	1	1	0	1	0	0	1	0	0	0	0

```
     <1     |  0  1  1  0    0  1  0  1    0  0  0  0
     <1     |  0  1  1  0    0  0  1  0    1  0  0  0
     <1     |  0  1  0  1    1  1  0  0    0  0  0  0
     <1     |  0  0  1  1    1  1  0  0    0  0  0  0
     <1     |  0  0  1  0    1  0  1  0    1  0  0  0
     <1     |  0  0  0  0    1  1  1  1    0  0  0  0
     <1     |  0  0  0  0    0  0  1  0    1  1  0  1
Missing 9 weeks:
      2     |  1  1  1  0    0  0  0  0    0  0  0  0
      1     |  1  1  0  1    0  0  0  0    0  0  0  0
     <1     |  1  1  0  0    1  0  0  0    0  0  0  0
     <1     |  1  1  0  0    0  0  1  0    0  0  0  0
     <1     |  1  1  0  0    0  0  0  0    0  0  1  0
      1     |  1  0  1  1    0  0  0  0    0  0  0  0
     <1     |  1  0  1  0    1  0  0  0    0  0  0  0
     <1     |  1  0  0  1    1  0  0  0    0  0  0  0
     <1     |  1  0  0  1    0  1  0  0    0  0  0  0
     <1     |  1  0  0  0    1  1  0  0    0  0  0  0
     <1     |  0  1  1  1    0  0  0  0    0  0  0  0
     <1     |  0  1  1  0    1  0  0  0    0  0  0  0
     <1     |  0  1  1  0    0  1  0  0    0  0  0  0
     <1     |  0  1  0  1    0  0  0  1    0  0  0  0
     <1     |  0  1  0  0    1  0  1  0    0  0  0  0
Missing 10 weeks:
      3     |  1  1  0  0    0  0  0  0    0  0  0  0
      1     |  1  0  1  0    0  0  0  0    0  0  0  0
     <1     |  1  0  0  1    0  0  0  0    0  0  0  0
     <1     |  1  0  0  0    0  1  0  0    0  0  0  0
      1     |  0  1  1  0    0  0  0  0    0  0  0  0
     <1     |  0  1  0  1    0  0  0  0    0  0  0  0
     <1     |  0  1  0  0    1  0  0  0    0  0  0  0
     <1     |  0  0  1  1    0  0  0  0    0  0  0  0
     <1     |  0  0  0  0    0  1  0  0    1  0  0  0
Missing 11 weeks:
      2     |  1  0  0  0    0  0  0  0    0  0  0  0
      3     |  0  1  0  0    0  0  0  0    0  0  0  0
     <1     |  0  0  1  0    0  0  0  0    0  0  0  0
     <1     |  0  0  0  1    0  0  0  0    0  0  0  0
     <1     |  0  0  0  0    0  0  0  1    0  0  0  0
     <1     |  0  0  0  0    0  0  0  0    0  0  0  1
Missing 12 weeks:
      5     |  0  0  0  0    0  0  0  0    0  0  0  0
------------+-----------------------------------------
    100%
```

Supplement K: Patterns of missing urine toxicology results for cocaine metabolites from week 1 to week 12 (a value of 0 indicates missing response in that week) in randomized controlled trials testing pharmacotherapies in people with cocaine use disorder.

.

Percent	Weeks 1	2	3	4	5	6	7	8	9	10	11	12
20%	1	1	1	1	1	1	1	1	1	1	1	1
Missing 1 week:												
<1	1	1	1	1	1	1	1	1	1	1	1	0
3	1	1	1	1	1	1	1	1	1	1	0	1
<1	1	1	1	1	1	1	1	1	1	0	1	1
<1	1	1	1	1	1	1	1	1	0	1	1	1
<1	1	1	1	1	1	1	1	0	1	1	1	1
<1	1	1	1	1	1	1	0	1	1	1	1	1
<1	1	1	1	1	1	0	1	1	1	1	1	1
<1	1	1	1	1	0	1	1	1	1	1	1	1
<1	1	1	1	0	1	1	1	1	1	1	1	1
<1	1	1	0	1	1	1	1	1	1	1	1	1
<1	1	0	1	1	1	1	1	1	1	1	1	1
<1	0	1	1	1	1	1	1	1	1	1	1	1
Missing 2 weeks:												
<1	1	1	1	1	1	1	1	1	1	1	0	0
1	1	1	1	1	1	1	1	1	1	0	1	0
1	1	1	1	1	1	1	1	1	1	0	0	1
<1	1	1	1	1	1	1	1	1	0	1	1	0
<1	1	1	1	1	1	1	1	1	0	1	0	1
<1	1	1	1	1	1	1	1	0	0	1	1	1
<1	1	1	1	1	1	1	0	1	1	1	1	0
<1	1	1	1	1	1	1	0	1	0	1	1	1
<1	1	1	1	1	1	1	0	0	1	1	1	1
<1	1	1	1	1	1	0	1	0	1	1	1	1
<1	1	1	1	1	0	0	1	1	1	1	1	1
<1	1	1	0	1	1	1	1	1	1	1	0	1
<1	1	1	0	1	1	1	1	1	1	0	1	1
Missing 3 weeks:												
5	1	1	1	1	1	1	1	1	1	0	0	0
<1	1	1	1	1	1	1	1	1	0	1	0	0
3	1	1	1	1	1	1	1	1	0	0	1	0
<1	1	1	1	1	1	1	1	1	0	0	0	1
<1	1	1	1	1	1	1	1	0	1	1	0	0
<1	1	1	1	1	1	1	0	0	1	0	1	1
<1	1	1	1	1	1	0	1	1	0	1	0	1
<1	1	1	1	1	0	1	1	1	1	1	0	0
<1	1	1	1	1	0	1	1	1	1	0	1	0
<1	1	1	1	1	0	1	0	1	0	1	1	1
<1	1	1	1	0	0	1	1	1	1	1	0	1
<1	1	1	0	1	1	1	1	1	1	1	0	0
Missing 4 weeks:												

```
      8     |  1  1  1  1   1  1  1  1   0  0  0  0
     <1     |  1  1  1  1   1  1  1  0   1  0  0  0
     <1     |  1  1  1  1   1  1  1  0   0  1  0  0
     <1     |  1  1  1  1   1  1  1  0   0  0  1  0
     <1     |  1  1  1  1   1  1  1  0   0  0  0  1
     <1     |  1  1  1  1   1  0  1  1   1  0  0  0
     <1     |  1  1  1  1   1  0  1  1   0  1  0  0
     <1     |  1  1  1  1   0  1  1  0   0  1  0  1
     <1     |  1  1  1  1   0  0  1  1   1  0  1  0
     <1     |  1  1  1  1   0  0  0  0   1  1  1  1
     <1     |  1  1  1  0   1  1  1  1   0  0  0  1
     <1     |  1  1  0  1   1  1  1  1   0  0  1  0
     <1     |  1  0  0  0   0  1  1  1   1  1  1  1
     <1     |  0  1  1  1   1  1  1  1   1  0  0  0
     <1     |  0  1  1  1   1  1  1  1   0  0  1  0
Missing 5 weeks:
      1     |  1  1  1  1   1  1  1  0   0  0  0  0
     <1     |  1  1  1  1   1  1  0  1   0  0  0  0
     <1     |  1  1  1  1   1  1  0  0   1  0  0  0
     <1     |  1  1  1  1   1  1  0  0   0  0  1  0
     <1     |  1  1  1  1   1  1  0  0   0  0  0  1
     <1     |  1  1  1  1   1  0  1  1   0  0  0  0
     <1     |  1  1  1  1   1  0  1  0   0  0  1  0
     <1     |  1  1  1  1   1  0  0  1   0  0  1  0
     <1     |  1  1  1  1   0  1  1  1   0  0  0  0
     <1     |  1  1  1  0   1  1  1  1   0  0  0  0
     <1     |  1  1  1  0   1  1  0  1   1  0  0  0
     <1     |  1  1  1  0   0  0  1  1   1  1  0  0
     <1     |  1  1  0  1   1  0  1  1   1  0  0  0
     <1     |  1  1  0  0   1  1  1  1   0  0  1  0
     <1     |  0  1  1  1   1  1  1  1   0  0  0  0
     <1     |  0  1  1  0   0  1  0  0   1  1  1  1
     <1     |  0  0  1  1   1  1  1  1   1  0  0  0
Missing 6 weeks:
      2     |  1  1  1  1   1  1  0  0   0  0  0  0
     <1     |  1  1  1  1   1  0  1  0   0  0  0  0
     <1     |  1  1  1  1   1  0  0  1   0  0  0  0
     <1     |  1  1  1  1   1  0  0  0   0  1  0  0
     <1     |  1  1  1  1   0  1  1  0   0  0  0  0
     <1     |  1  1  1  1   0  1  0  0   0  0  0  1
     <1     |  1  1  1  1   0  0  0  0   1  0  1  0
     <1     |  1  1  0  1   1  1  1  0   0  0  0  0
     <1     |  1  1  0  1   0  1  0  0   0  1  0  1
     <1     |  1  1  0  0   0  1  1  1   0  0  1  0
     <1     |  1  0  0  1   1  1  1  1   0  0  0  0
Missing 7 weeks:
      1     |  1  1  1  1   1  0  0  0   0  0  0  0
     <1     |  1  1  1  1   0  1  0  0   0  0  0  0
     <1     |  1  1  1  1   0  0  0  1   0  0  0  0
     <1     |  1  1  1  1   0  0  0  0   1  0  0  0
     <1     |  1  1  1  0   0  1  0  0   0  0  0  1
     <1     |  1  1  1  0   0  0  1  0   1  0  0  0
```

```
     <1     |  1  1  0  1    1  1  0  0    0  0  0  0
     <1     |  1  1  0  0    1  1  1  0    0  0  0  0
     <1     |  1  0  1  1    1  1  0  0    0  0  0  0
     <1     |  0  1  1  1    1  0  1  0    0  0  0  0
     <1     |  0  1  1  1    1  0  0  1    0  0  0  0
     <1     |  0  1  1  0    0  0  0  1    0  1  1  0
     <1     |  0  0  1  0    0  1  1  1    1  0  0  0
Missing 8 weeks:
      2     |  1  1  1  1    0  0  0  0    0  0  0  0
     <1     |  1  1  1  0    1  0  0  0    0  0  0  0
     <1     |  1  1  1  0    0  1  0  0    0  0  0  0
     <1     |  1  1  0  1    1  0  0  0    0  0  0  0
     <1     |  1  1  0  0    1  1  0  0    0  0  0  0
     <1     |  1  0  1  0    0  1  1  0    0  0  0  0
     <1     |  1  0  0  1    1  0  0  1    0  0  0  0
Missing 9 weeks:
      3     |  1  1  1  0    0  0  0  0    0  0  0  0
     <1     |  1  1  0  1    0  0  0  0    0  0  0  0
     <1     |  1  1  0  0    1  0  0  0    0  0  0  0
     <1     |  1  1  0  0    0  1  0  0    0  0  0  0
     <1     |  1  1  0  0    0  0  1  0    0  0  0  0
     <1     |  1  0  1  1    0  0  0  0    0  0  0  0
     <1     |  0  1  1  1    0  0  0  0    0  0  0  0
     <1     |  0  1  1  0    0  0  0  0    0  0  0  1
Missing 10 weeks:
      3     |  1  1  0  0    0  0  0  0    0  0  0  0
     <1     |  1  0  1  0    0  0  0  0    0  0  0  0
     <1     |  1  0  0  1    0  0  0  0    0  0  0  0
     <1     |  1  0  0  0    0  0  0  0    0  0  1  0
     <1     |  0  1  1  0    0  0  0  0    0  0  0  0
     <1     |  0  1  0  1    0  0  0  0    0  0  0  0
     <1     |  0  0  0  0    1  0  0  0    1  0  0  0
Missing 11 weeks:
      2     |  1  0  0  0    0  0  0  0    0  0  0  0
     <1     |  0  1  0  0    0  0  0  0    0  0  0  0
Missing 12 weeks:
     25     |  0  0  0  0    0  0  0  0    0  0  0  0
------------+-------------------------------------------
    100%
```

Supplement L: Patterns of missing self-reports on cocaine use from week 1 to week 12 (a value of 0 indicates missing response in that week) in randomized controlled trials testing pharmacotherapies in people with cocaine use disorder.

	Weeks											
Percent	1	2	3	4	5	6	7	8	9	10	11	12
40%	1	1	1	1	1	1	1	1	1	1	1	1
Missing 1 week:												
4	1	1	1	1	1	1	1	1	1	1	1	0
<1	1	1	1	1	1	1	1	1	1	1	0	1
<1	1	1	1	1	1	1	0	1	1	1	1	1
<1	1	1	1	1	0	1	1	1	1	1	1	1
Missing 2 weeks:												
1	1	1	1	1	1	1	1	1	1	1	0	0
<1	1	1	1	1	1	1	1	1	1	0	1	0
1	1	1	1	1	1	1	1	1	1	0	0	1
<1	1	1	1	1	1	1	0	1	0	1	1	1
<1	1	1	1	1	0	0	1	1	1	1	1	1
<1	1	0	0	1	1	1	1	1	1	1	1	1
<1	0	1	1	1	1	1	1	1	1	1	1	0
Missing 3 weeks:												
5	1	1	1	1	1	1	1	1	1	0	0	0
<1	1	1	1	1	1	1	1	1	0	1	0	0
<1	1	1	1	1	1	1	1	1	0	0	1	0
3	1	1	1	1	1	1	1	1	0	0	0	1
<1	1	1	1	1	1	1	1	0	1	1	0	0
<1	1	1	1	1	1	1	1	0	0	0	1	1
<1	1	1	1	1	1	0	0	0	1	1	1	1
<1	1	1	1	1	0	1	1	1	1	1	0	0
Missing 4 weeks:												
12	1	1	1	1	1	1	1	1	0	0	0	0
<1	1	1	1	1	1	1	1	0	0	0	1	0
<1	1	1	1	1	1	1	1	0	0	0	0	1
<1	1	1	1	1	1	0	0	0	0	1	1	1
<1	1	1	0	1	1	0	1	1	0	0	1	1
Missing 5 weeks:												
3	1	1	1	1	1	1	1	0	0	0	0	0
<1	1	1	1	1	1	1	0	0	0	0	1	0
<1	1	1	1	1	1	1	0	0	0	0	0	1
<1	1	1	1	1	1	0	1	1	0	0	0	0
<1	1	1	1	1	1	0	0	1	1	0	0	0
<1	1	1	1	1	1	0	0	0	0	0	1	1
<1	1	1	1	1	0	0	0	1	1	1	0	0
<1	1	1	1	0	1	1	0	0	1	1	0	0
<1	1	1	1	0	0	1	1	1	0	0	0	1
<1	1	1	1	0	0	0	1	1	1	1	0	0
<1	1	1	0	0	1	1	1	1	0	0	0	1
Missing 6 weeks:												
3	1	1	1	1	1	1	0	0	0	0	0	0

```
     <1     |  1  1  1  1    1  0  0  1    0  0  0  0
     <1     |  1  1  1  1    1  0  0  0    0  1  0  0
     <1     |  0  1  1  1    1  1  1  0    0  0  0  0
Missing 7 weeks:
      2     |  1  1  1  1    1  0  0  0    0  0  0  0
     <1     |  1  1  1  1    0  0  0  0    1  0  0  0
     <1     |  1  1  1  1    0  0  0  0    0  1  0  0
     <1     |  1  1  1  0    1  1  0  0    0  0  0  0
     <1     |  1  1  0  0    1  1  1  0    0  0  0  0
Missing 8 weeks:
      4     |  1  1  1  1    0  0  0  0    0  0  0  0
     <1     |  1  1  1  0    0  0  0  0    0  0  0  1
     <1     |  1  1  0  1    1  0  0  0    0  0  0  0
     <1     |  0  1  1  1    1  0  0  0    0  0  0  0
Missing 9 weeks:
      5     |  1  1  1  0    0  0  0  0    0  0  0  0
     <1     |  1  0  1  1    0  0  0  0    0  0  0  0
Missing 10 weeks:
      4     |  1  1  0  0    0  0  0  0    0  0  0  0
Missing 11 weeks:
      4     |  1  0  0  0    0  0  0  0    0  0  0  0
     <1     |  0  1  0  0    0  0  0  0    0  0  0  0
     <1     |  0  0  0  1    0  0  0  0    0  0  0  0
Missing 12 weeks:
      2     |  0  0  0  0    0  0  0  0    0  0  0  0
------------+--------------------------------------
    100%
```

Supplement M: Model fit indices for multi-trajectory analysis of missing data on craving, urine toxicology for cocaine and self-reported cocaine use in randomized controlled trials testing pharmacotherapies in people with cocaine use disorder.

Model	Estimated group sizes	BIC (n=1,019)[a]	BIC (n=25,890)[b]	AIC	Relative entropy
1-group	G1:100%	-22758	-22774	-22735	--
2-group	G1:71.7%<br>G2:28.3%	-17107	-17141	-17059	0.99
3-group	G1:56.3%<br>G2:15.3%<br>G3:28.4%	-14168	-14220	-14095	0.99
4-group	G1:29.4%<br>G2:31.6%<br>G3:22.3%<br>G4:16.7%	-12370	-12440	-12273	0.98
5-group	G1:30.1%<br>G2:24.9%<br>G3:14.7%<br>G4:16.6%<br>G5:13.7%	-11772	-11859	-11650	0.98
6-group	--[c]	--[c]	--[c]	--[c]	--[c]

Abbreviations: BIC: Bayesian Information Criterion; AIC: Akaike Information Criterion; APP: Average Posterior Probability; G1 through G5: trajectory groups 1 through 5.

a. Person-level BIC.
b. Assessment-level BIC.
c. The model did not converge.

Supplement N: Trajectory groups in multi-trajectory analysis of missing data on craving, urine toxicology (Utox) for cocaine and self-reported cocaine use in the 5-group model in randomized controlled trials testing pharmacotherapies in people with cocaine use disorder.

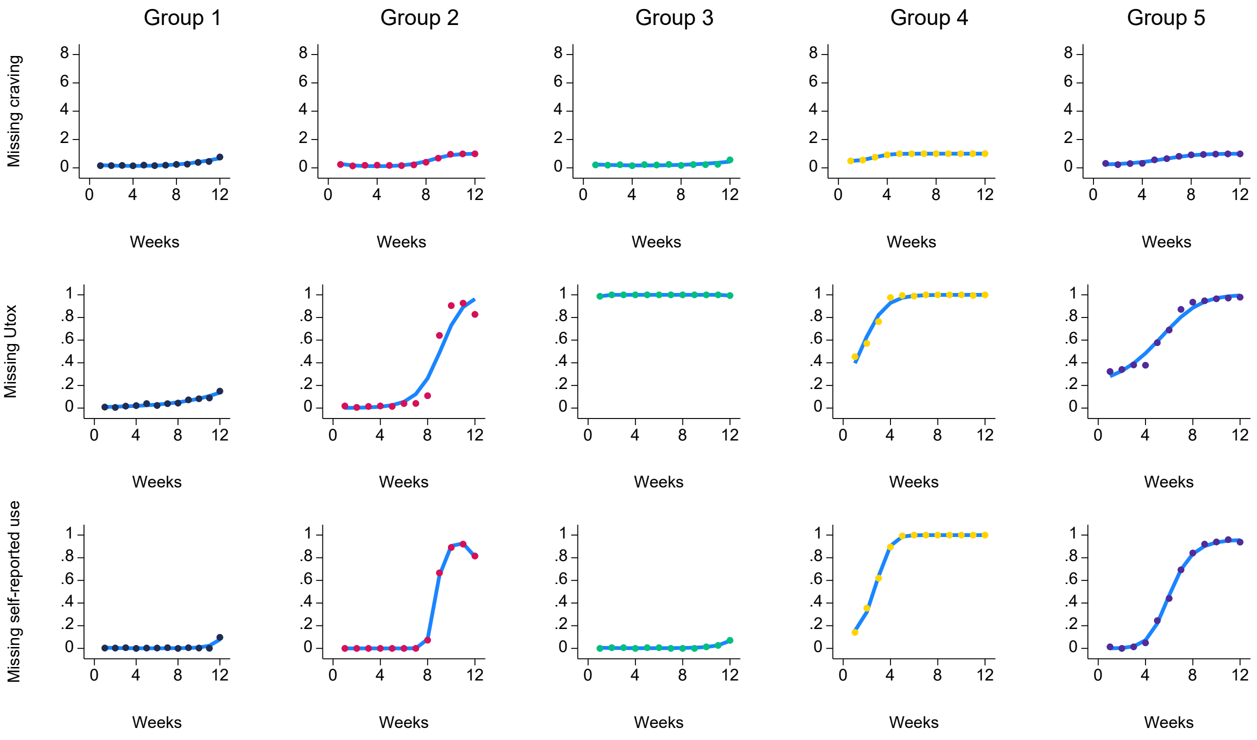

Supplement O: Association of missing data trajectories with the groups obtained in the multi-trajectory analysis in randomized controlled trials testing pharmacotherapies in people with cocaine use disorder. Data are reported separately for all trials the 12-week trials.

Missing data trajectory groups	Groups in the 3-group multi-trajectory model for the outcomes							
	All trials[a]				12-week trials[b]			
	Group 1	Group 2	Group 3	Total	Group 1	Group 2	Group 3	Total
	%	%	%	%	%	%	%	%
Group 1	31.5	31.7	27.2	30.6	42.5	39.2	31.9	38.5
Group 2	28.8	22.8	21.2	24.5	3.9	4.2	7.1	4.8
Group 3	18.0	11.2	16.0	14.7	32.6	20.8	27.0	25.7
Group 4	10.8	19.4	20.4	16.6	10.5	20.8	19.2	17.7
Group 5	11.0	15.0	15.2	13.6	10.5	14.9	14.9	13.6
Total	100.0	100.0	100.0	100.0	100.0	100.0	100.0	100.0

a. Test of association of missing trajectory groups with multi-trajectory groups of craving and drug use for all trials: Chi-squared=27.36, df=8, p=0.001.

b. Test of association of missing trajectory groups with multi-trajectory groups of craving and drug use for 12-weeks trials: Chi-squared=19.60, df=8, p=0.012.

Supplement P: Trajectory groups in multi-trajectory analysis of cocaine craving, urine-toxicology (Utox) ascertained cocaine use and self-reported cocaine use in the 3-group model adjusting for missing data trajectories in randomized controlled trials testing pharmacotherapies in people with cocaine use disorder.

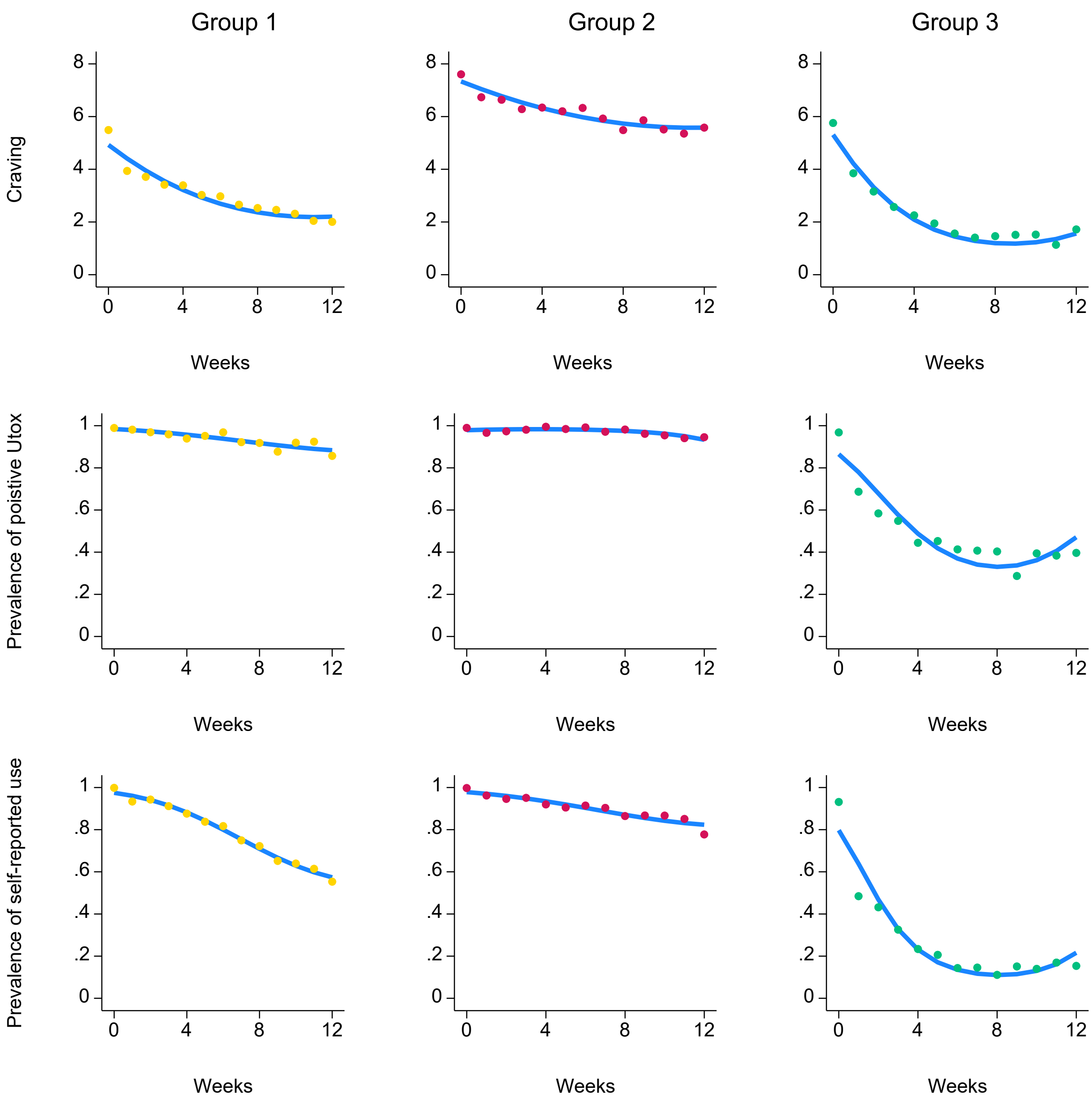

Supplement Q: Correspondence between trajectory groups in the main analysis (unadjusted for missing data) and trajectory groups from analysis adjusting for missing data trajectories in randomized controlled trials testing pharmacotherapies in people with cocaine use disorder. Highlighted cells indicate the same trajectory groups in the main analysis and in the model adjusting for missing data.

Trajectory groups in the main analysis (unadjusted)	Trajectory groups after adjusting for missing data			
	Group 1	Group 2	Group 3	Total
	n (row %)	n (row %)	n (row %)	n (row %)
1	330 (88.7)	25 (6.7)	17 (4.6)	372 (100.0)
2	1 (0.2)	447 (99.8)	0 (0.0)	448 (100.0)
3	1 (0.4)	0 (0.0)	249 (99.6)	250 (100.0)
Total	332 (31.0)	472 (44.1)	266 (24.9)	1,070 (100.0)

Supplement R: Trajectory groups in multi-trajectory analysis of cocaine craving, urine-toxicology (Utox) ascertained cocaine use and self-reported cocaine use in the 3-group model based on 12-week randomized controlled trials testing pharmacotherapies in people with cocaine use disorder.

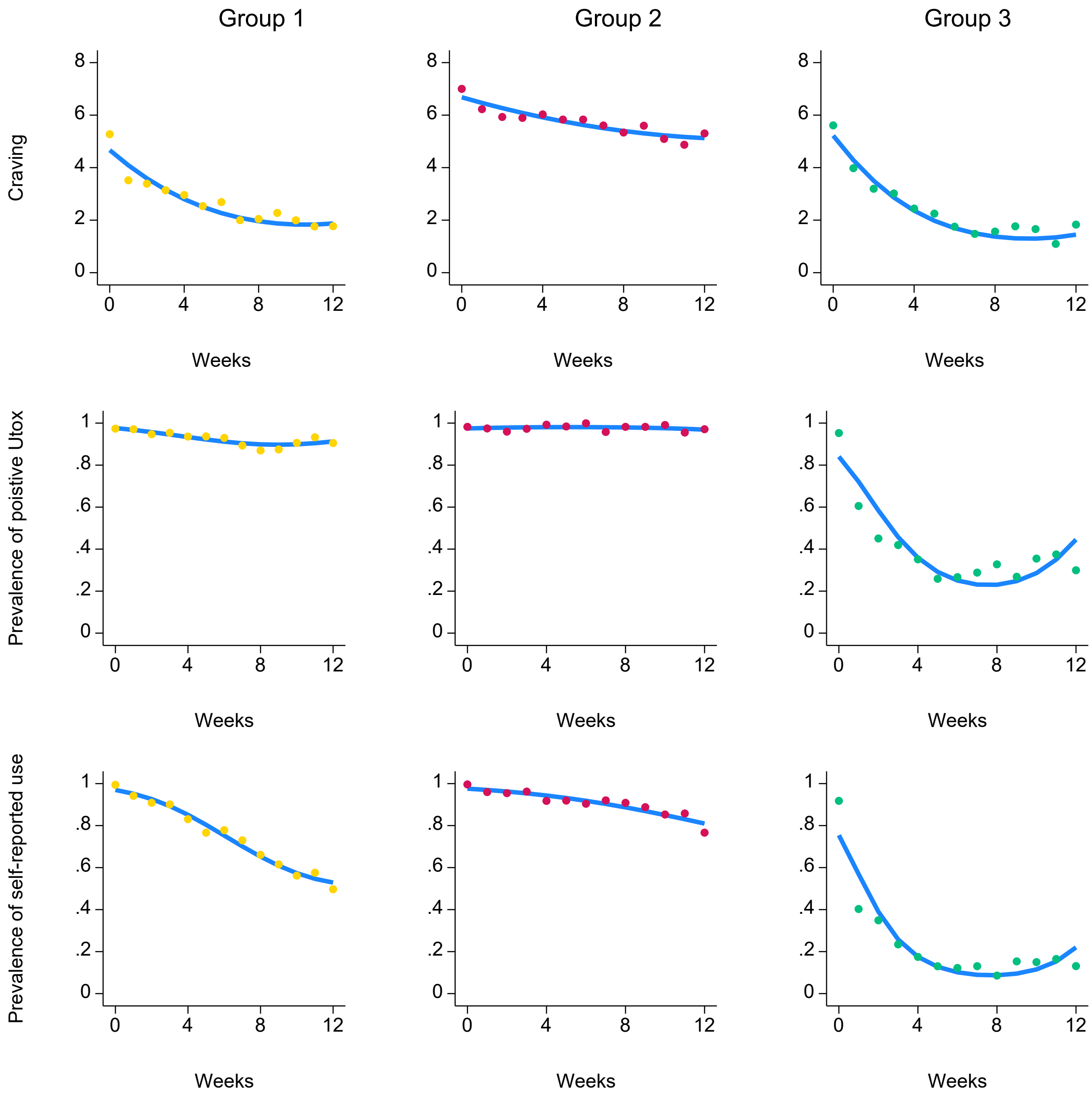

Supplement S: Model fit indices for multi-trajectory analysis of craving and urine toxicology for cocaine (2 variables) in randomized controlled trials testing pharmacotherapies in people with cocaine use disorder.

**Model**	**Estimated group sizes**	**BIC (n=1,019)[a]**	**BIC (n=15,604)[b]**	**AIC**	**Relative entropy**
1-group	G1:100%	-20609	-20618	-20592	--
2-group	G1:63.3%<br>G2:36.7%	-19070	-19090	-19032	0.80
3-group	G1:41.1%<br>G2:30.0%<br>G3:28.9%	-18558	-18589	-18501	0.77
4-group	G1:40.6%<br>G2:30.8%<br>G3:27.9%<br>G4:0.6%	-18544	-18586	-18467	0.81

Abbreviations: BIC: Bayesian Information Criterion; AIC: Akaike Information Criterion; APP: Average Posterior Probability; and G1 through G4: trajectory groups 1 through 4.

a. Person-level BIC.

b. Assessment-level BIC.

Supplement T: Trajectory groups in multi-trajectory analysis of cocaine craving and urine-toxicology for cocaine (Utox) (2 variables) in the 3-group model in randomized controlled trials testing pharmacotherapies in people with cocaine use disorder.

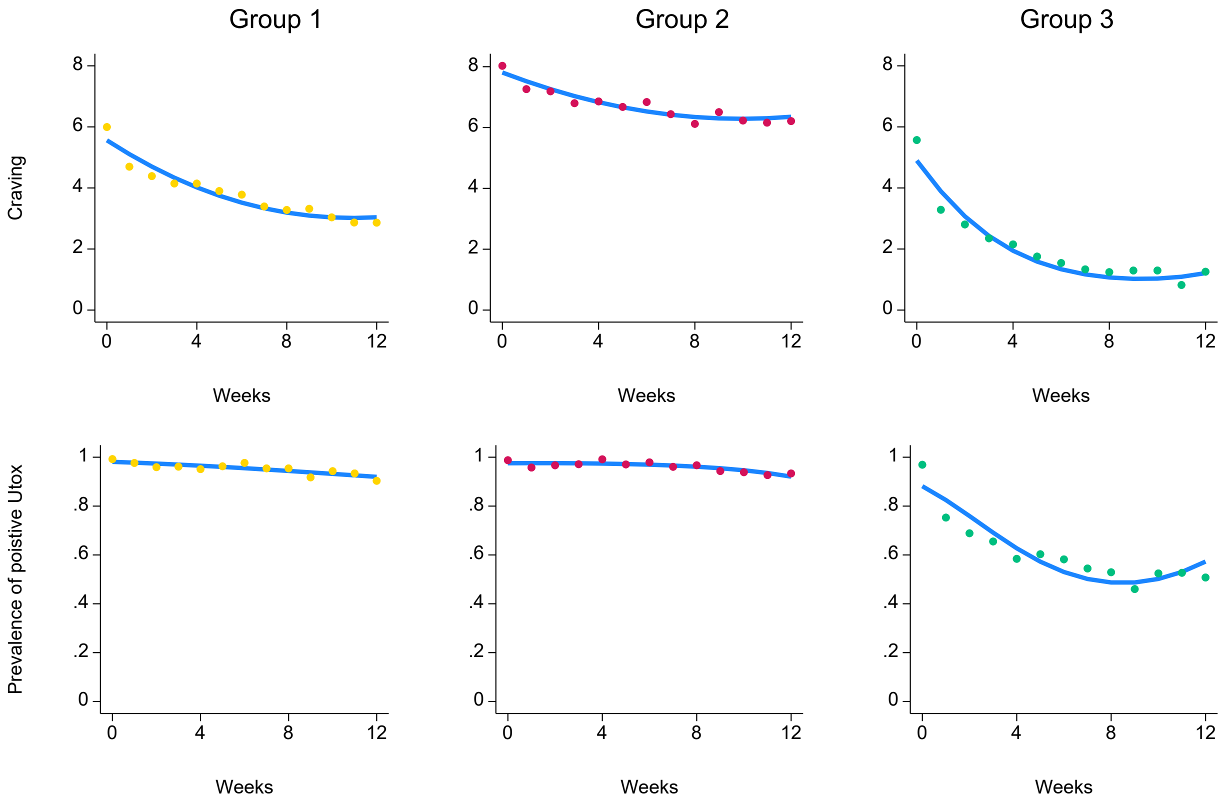

References

Anderson, A.L., Reid, M.S., Li, S.H., Holmes, T., Shemanski, L., Slee, A., Smith, E.V., Kahn, R., Chiang, N., Vocci, F., Ciraulo, D., Dackis, C., Roache, J.D., Salloum, I.M., Somoza, E., Urschel, H.C., 3rd, Elkashef, A.M., 2009. Modafinil for the treatment of cocaine dependence. Drug Alcohol Depend 104(1-2), 133-139.

Elkashef, A., Fudala, P.J., Gorgon, L., Li, S.H., Kahn, R., Chiang, N., Vocci, F., Collins, J., Jones, K., Boardman, K., Sather, M., 2006. Double-blind, placebo-controlled trial of selegiline transdermal system (STS) for the treatment of cocaine dependence. Drug Alcohol Depend 85(3), 191-197.

Kahn, R., Biswas, K., Childress, A.R., Shoptaw, S., Fudala, P.J., Gorgon, L., Montoya, I., Collins, J., McSherry, F., Li, S.H., Chiang, N., Alathari, H., Watson, D., Liberto, J., Beresford, T., Stock, C., Wallace, C., Gruber, V., Elkashef, A., 2009. Multi-center trial of baclofen for abstinence initiation in severe cocaine-dependent individuals. Drug Alcohol Depend 103(1-2), 59-64.

Shoptow, S., Newton, T., Rawson, R.A., Ling, W., Kobahigawa, J., 2017. Phase 2, double-blind, placebo controlled trial of cabergoline for the treatment of cocaine dependence. ClinicalTrials.gov. National Library of Medicine. https://clinicaltrials.gov/study/NCT00033111. (Accessed: August 7, 2025).

Winhusen, T., Somoza, E., Ciraulo, D.A., Harrer, J.M., Goldsmith, R.J., Grabowski, J., Coleman, F.S., Mindrum, G., Kahn, R., Osman, S., Mezinskis, J., Li, S.H., Lewis, D., Horn, P., Montgomery, M.A., Elkashef, A., 2007a. A double-blind, placebo-controlled trial of tiagabine for the treatment of cocaine dependence. Drug Alcohol Depend 91(2-3), 141-148.

Winhusen, T., Somoza, E., Sarid-Segal, O., Goldsmith, R.J., Harrer, J.M., Coleman, F.S., Kahn, R., Osman, S., Mezinskis, J., Li, S.H., Lewis, D., Afshar, M., Ciraulo, D.A., Horn, P., Montgomery, M.A., Elkashef, A., 2007b. A double-blind, placebo-controlled trial of reserpine for the treatment of cocaine dependence. Drug Alcohol Depend 91(2-3), 205-212.
